# A high-resolution genotype–phenotype map identifies the *TaSPL17* controlling grain number and size in wheat

**DOI:** 10.1186/s13059-023-03044-2

**Published:** 2023-08-28

**Authors:** Yangyang Liu, Jun Chen, Changbin Yin, Ziying Wang, He Wu, Kuocheng Shen, Zhiliang Zhang, Lipeng Kang, Song Xu, Aoyue Bi, Xuebo Zhao, Daxing Xu, Zhonghu He, Xueyong Zhang, Chenyang Hao, Jianhui Wu, Yan Gong, Xuchang Yu, Zhiwen Sun, Botao Ye, Danni Liu, Lili Zhang, Liping Shen, Yuanfeng Hao, Youzhi Ma, Fei Lu, Zifeng Guo

**Affiliations:** 1 Key Laboratory of Plant Molecular Physiology, Institute of Botany, Chinese Academy of Sciences, Beijing, 100093 China; 2 University of Chinese Academy of Sciences, Beijing, 100049 China; 3 Institute of Crop Sciences, Chinese Academy of Agricultural Sciences (CAAS), Beijing, 100081 China; 4 State Key Laboratory of Plant Cell and Chromosome Engineering, Institute of Genetics and Developmental Biology, Innovative Academy of Seed Design, Chinese Academy of Sciences, Beijing, 10011 China; 5 International Maize and Wheat Improvement Center (CIMMYT) China Office, c/o CAAS, Beijing, 100081 China; 6 State Key Laboratory of Crop Stress Biology for Arid Areas, Northwest A&F University, Yangling, 712100 Shaanxi China; 7 CAS-JIC Centre of Excellence for Plant and Microbial Science (CEPAMS), Institute of Genetics and Developmental Biology, Chinese Academy of Sciences, Beijing, 100093 China

**Keywords:** Grain number, Grain size, GWAS, *TaSPL17*, Wheat

## Abstract

**Background:**

Large-scale genotype–phenotype association studies of crop germplasm are important for identifying alleles associated with favorable traits. The limited number of single-nucleotide polymorphisms (SNPs) in most wheat genome-wide association studies (GWASs) restricts their power to detect marker-trait associations. Additionally, only a few genes regulating grain number per spikelet have been reported due to sensitivity of this trait to variable environments.

**Results:**

We perform a large-scale GWAS using approximately 40 million filtered SNPs for 27 spike morphology traits. We detect 132,086 significant marker-trait associations and the associated SNP markers are located within 590 associated peaks. We detect additional and stronger peaks by dividing spike morphology into sub-traits relative to GWAS results of spike morphology traits. We propose that the genetic dissection of spike morphology is a powerful strategy to detect signals for grain yield traits in wheat. The GWAS results reveal that *TaSPL17* positively controls grain size and number by regulating spikelet and floret meristem development, which in turn leads to enhanced grain yield per plant. The haplotypes at *TaSPL17* indicate geographical differentiation, domestication effects, and breeding selection.

**Conclusion:**

Our study provides valuable resources for genetic improvement of spike morphology and a fast-forward genetic solution for candidate gene detection and cloning in wheat.

**Supplementary Information:**

The online version contains supplementary material available at 10.1186/s13059-023-03044-2.

## Background

Global cereal production should increase by around 50% during the first half of the twenty-first century to satisfy the expected rising demand driven by population growth [1]. Wheat (*Triticum aestivum* L.) is one of the most important crops worldwide. It supports more than 2.5 billion people [2] and accounts for around 20% of dietary calories and protein consumed by humankind [3], around 30% of global grain production, and around 45% of cereals used as food [4]. Since crop diseases, climate change, and abiotic stresses (e.g., drought and heat) cause significant grain yield loss, raising grain yield remains the primary goal in most wheat-producing areas [5–7].

Wheat spikes are the organs that determine grain yield. Wheat spikes consist of spikelets that produce reproductive structures called florets. An individual floret consists of lemma, palea, anthers, carpels, awns, and lodicules. Increased partitioning of assimilates to grains has a great influence on grain yield in wheat. Indeed, assimilate distribution between grains and spike chaff plays a critical role in determining grain weight in wheat [8–10]. However, the genetic connections between grains and other spike components (spike chaff) are largely unknown. A greater and global understanding of the genetic relationships between grains and spike chaff is critical for achieving desired gains in specific traits by harnessing different allelic combinations.

Genome-wide association study (GWAS) is a powerful strategy for dissecting the genetic basis of complex traits and identifying marker–trait associations [10–12]. Although numerous wheat GWAS have been conducted, the limited number of single-nucleotide polymorphisms (SNPs) (< 1 million) used for these studies limit their ability to detect marker-trait associations. Due to the large genome size and high proportion of repetitive sequences in hexaploid wheat, progress in whole-genome sequencing is relatively slow, restricting wheat research. From 2012 to 2018, great strides have been achieved in genome sequencing of diploid, tetraploid, and hexaploid wheat [13–19]. The genome sequence of hexaploid wheat was released in 2018 (IWGSC, 2018), which greatly increased the number of SNPs available for investigation, revealing much about wheat evolution and domestication [20–25].

Although several studies identifying quantitative trait locus (QTLs) associated with spike morphology traits have been reported, few studies have focused on more than ten traits in a single study. Here, we conducted a large-scale GWAS based on approximately 40 million filtered SNPs for 27 spike morphology traits to dissect and understand the genetic basis of spike morphology. This approach may save a lot of time and work to get relatively precise locations of candidate genes leading to fast cloning of those genes for economically important traits. The genotype-phenotype map of these 27 traits revealed novel genomic regions associated with each putative trait and candidate genes. The 27 spike morphology traits enabled us to identify 392 additional genomic regions, indicating the value of dissecting spike morphology genetics for the detection of novel signals. Through GWAS, we identified and validated the gene *SQUAMOSA PROMOTER BINDING PROTEIN-LIKE17* (*TaSPL17*), whose product positively regulates grain yield through grain number and size. Our results provide insights into the genetic basis of wheat spike morphology traits and serve as a resource for the genetic improvement of wheat grain yield.

## Results

### Genetic variation and population structure

In this study, we used genotype data from wheat accessions to create a whole-genome genetic variation map of wheat [20, 26]. The latest version of VMap (VMap 2.0) is based on 1062 wheat accessions with multiple ploidy levels [27]. From these, we selected 306 hexaploid wheat worldwide accessions [28]. High-coverage whole-genome sequencing (~ 10 ×) enabled the high-confidence identification of 117,398,144 SNPs in the 306 wheat accessions; the false-positive error rate of variant calling (i.e., the proportion of segregating sites in the reference accession Chinese Spring) was only 0.00067%. We used 40,710,923 filtered SNPs (A genome, 17,509,041; B genome, 20,603,513; D genome, 2,598,369) with a minor allele frequency (MAF) of at least 0.05 for this study.

**Fig. 1:**
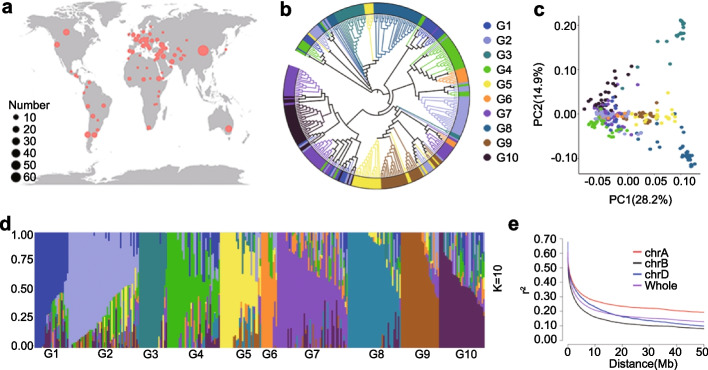
Diversity represented by 306 hexaploid wheat accessions.** a** Geographical distribution of wheat accessions, as indicated by circles on the world map. Circle size is proportional to the number of accessions. **b** Phylogenetic tree of all accessions calculated from whole-genome SNPs. The colors indicate the 10 groups (G1-G10). **c** Principal component analysis (PCA) plot of the first two components (PC1 and PC2) of the 306 wheat accessions. **d** Population structure analysis with *K* = 10. The *y*-axis quantifies cluster membership, and the *x*-axis represents the different accessions. The order and position of the accessions along the *x*-axis are consistent with those in the phylogenetic tree. **e** Decay of linkage disequilibrium (LD) in the wheat genome. LD decay was estimated based on SNP markers from all three genomes: A genome (chr A), B genome (chr B), and D genome (chr D)

These 306 wheat accessions were mainly landraces collected from more than 70 countries (Fig. 1a, Additional file 1: Table S1). We constructed a phylogenetic tree (Fig. 1b), performed a principal component analysis (PCA) (Fig. 1c) and a population structure analysis (Fig. 1d) on these 306 accessions. The PCA results demonstrated that geography at the continental scale is the most important correlate of genetic structure. We classified the accessions into 10 genetic groups, G1–G10, comprising 23, 48, 19, 36, 28, 11, 48, 36, 26, and 31 samples, respectively (Additional file 1: Table S2). The first and second principal components (PC1 and PC2) of the PCA explained 28.2% and 14.9% of the standing variance, respectively, and reflected geographical factors, with PC1 separating European (G2) and Asian (G8) wheat accessions, and PC2 distinguishing between Middle Eastern (G5) and African (G9) wheat accessions (Fig. 1c, Additional file 1: Tables S1 and S2). These groups were consistent with the related phylogenetic tree. As *K* increased in the population structure analysis, the corresponding cross-validation error decreased and reached a trough at *K* = 10 (Additional file 2: Figure S1). We also calculated the decay rate of linkage disequilibrium (LD) as the pairwise correlation coefficient (*r*^*2*^) from the maximum value to the half maximum, which reached 3.08 Mb, for all accessions with variation among different genomes (Fig. 1e, Additional file 1: Table S3). LD in the A, B, and D genomes was 6.04, 2.39, and 3.03 Mb, respectively.

### Overview of spike morphology traits

Previous work has focused on grain number and size to identify quantitative trait locus (QTLs) and other genomic regions related to grain yield [29]. Here, we looked for genomic signals in grain yield traits by dissecting the genetic connections between grains and other spike components (spike chaff), because assimilate partitioning is closely associated with spike morphology and grain yield traits in wheat. We thus dissected grain weight, number, and size using the traits associated with assimilate distribution between spike components. We analyzed a total of 27 spike morphology traits corrected on 306 worldwide wheat accessions. These 27 traits comprised 12 measured traits: spike length, spike dry weight, grain weight per spike, awn weight per spike, grain number per spike, spikelet number per spike, spike chaff weight per spike, rachis weight per spike, individual spikelet weight, grain number per spikelet, chaff weight per spikelet, and grain weight per spikelet (Additional file 1: Tables S4, S5 and S6). In addition, we used the 12 measured traits to determine 15 ratios/traits to estimate the distribution of assimilates among spike components (Additional file 1: Tables S4, S5 and S6). The 306 accessions displayed strong variation for all 27 traits (Additional file 1: Tables S4, S5 and S6, Additional file 2: Figures S2 and S3). We estimated the broad sense heritability (*h*^*2*^) for all 27 traits by calculating repeatability between raw phenotypes. Generally, direct traits exhibited high heritability, whereas the heritability of the indirect traits (the ratios between spike direct traits) was relatively low (Additional file 1: Table S4).

All traits followed a normal distribution with three exceptions: awn weight per spike, awn weight/grain weight, and awn weight/spike chaff weight (Additional file 2: Figure S3). The abnormal distribution of these traits may be attributed to the geographical differentiation of wheat awn. Interestingly, these three traits had relatively high heritability among the 27 traits (awn weight/spike weight, *h*^*2*^ = 0.87; awn weight/grain weight, *h*^*2*^ = 0.73; awn weight/spike chaff weight, *h*^*2*^ = 0.72) (Additional file 1: Table S4). The broad genotypic variation and relatively high heritability of most traits (Additional file 1: Tables S4, S5 and S6) suggest the large potential and a genetic basis for the increase in grain yield by manipulating spike morphology.

The strong genetic effects of spike length, spikelet number per spike, awn weight per spike, and spike density according to an analysis of variance (ANOVA) analysis were consistent with their relatively high heritability (Additional file 1: Tables S4, S5 and S6). The environmental effects and the influence of genotype × environment were significant for grain number and thousand kernel weight (spikelet), which may explain their low heritability (Additional file 1: Tables S4, S5 and S6). The large residuals of spike chaff weight, chaff weight per spikelet, grain weight/chaff weight, grain weight per spikelet /chaff weight(spikelet), grain weight per spikelet /spikelet weight (spikelet), and chaff weight per spikelet /spikelet weight indicate that the dissection of these traits not only magnified the differences between genotypes, but also led to the strong variations among replicates within individual genotype, which resulted in low heritability (Additional file 1: Tables S4, S5 and S6).

The high-resolution dissection of spike morphology above captured several important relationships (Additional file 2: Figure S4). For instance, grain weight per spikelet/spikelet weight and chaff weight/spikelet weight were strongly and negatively correlated. Consistently, grain weight per spike/spike weight exhibited close and negative connections with spike chaff weight/spike weight. Awn weight per spike was strongly and positively associated with spike chaff weight, suggesting that awn is an important part of spike chaff.

### GWAS for 27 spike morphology traits

**Fig. 2:**
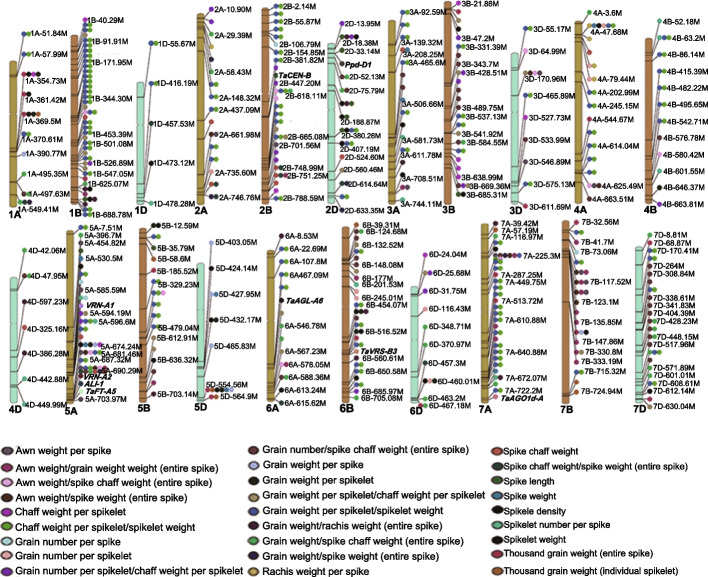
Genotype–phenotype map for the 27 spike morphology traits. Genotype–phenotype map with the identified genomic regions for the corresponding traits in each chromosome were aligned to the reference sequence of bread wheat. Traits determined based on the entire spike are indicated where applicable. Individual spikelets were collected from the center of spike. The symbol “/” indicates the ratio between the two traits

With the set of 40,710,923 SNPs defined above (MAF > 0.05; missing rate < 20%; missing genotype rate < 0.1), we performed GWAS for 27 spike morphology traits. We identified 132,086 significant marker–trait associations (-log_10_[*P* value] > 5.00) across the three subgenomes, with 33,619 associations from the A genome, 90,158 from the B genome, and 8309 for the D genome (Additional file 1: Table S7). We also used LD and connections between markers to delineate 590 genomic regions associated with the 27 traits, comprised of 216 for the A genome, 254 for the B genome, and 120 for the D genome (Fig. 2, Additional file 1: Table S8). Of the 590 identified genomic regions, 422 were associated with more than one trait. Some associated genomic regions exhibited pleiotropic effects on the traits related to grain number/size and assimilate partitioning, while the genomic regions for grain number/size traits were not as strong as the regions for assimilate partitioning traits. For instance, we identified one region (1B: 628,535,896-629,920,890) when mapping for grain weight/rachis weight (entire spike) and spikelet density. The best SNP for the signal of grain weight/rachis weight (entire spike) (assimilate partitioning) were of much higher significance (- log_10_ [*P* value] = 9.37) than the best SNP of spikelet density (-log_10_ [*P* value] = 6.07). In addition, 392 peaks associated with assimilate distribution (ratios between spike component weight) were not observed for the remaining spike morphology/grain yield traits (Additional file 1: Tables S8 and S9). Haplotype analyses showed that 296 of 392 peaks have effects on the remaining spike morphology/grain yield traits (Additional file 1: Table S9). These results suggest that the ratios between the weights of spike components minimize the variance among the values of spike components, which in turn maximize the ability to detect differences for that spike component. This approach increases the power of GWAS to detect significant associations and highlights the necessity of conducting GWAS on assimilate partitioning traits.

We included awn weight as an internal control for GWAS. *Awn Length Inhibitor 1* (*ALI-1*, *TraesCS5A02G542800*) was previously confirmed as the causal gene for the *Tipped1* (*B1*) locus that controls awn initiation and development [30–32]. We detected 1325 marker-trait associations of awn weight, within the region (5A:690,292,073-703,437,181), which includes *ALI-1* (*TraesCS5A02G542800*) (Additional file 1: Table S8, Additional file 2: Figure S5). In addition, we identified *VERNALIZATION-A1* (*VRN-A1*), *VRN-A2*, *Photoperiod-D1* (*Ppd-D1*), *FLOWERING LOCUS T-A5* (*TaFT-A5*)*, AGAMOUS-LIKE 6* (*TaAGL-A6*), *ARGONAUTE 1d* (*TaAGO1d-A*), *SIX-ROWED SPIKE-B3* (*TaVRS-B3*), and *CENTRORADIALIS-B* (*TaCEN-B*) as candidate genes for spike morphology traits (Fig. 2, Additional file 1: Table S8).

### GWAS identification of a candidate gene regulating grain number and size

**Fig. 3:**
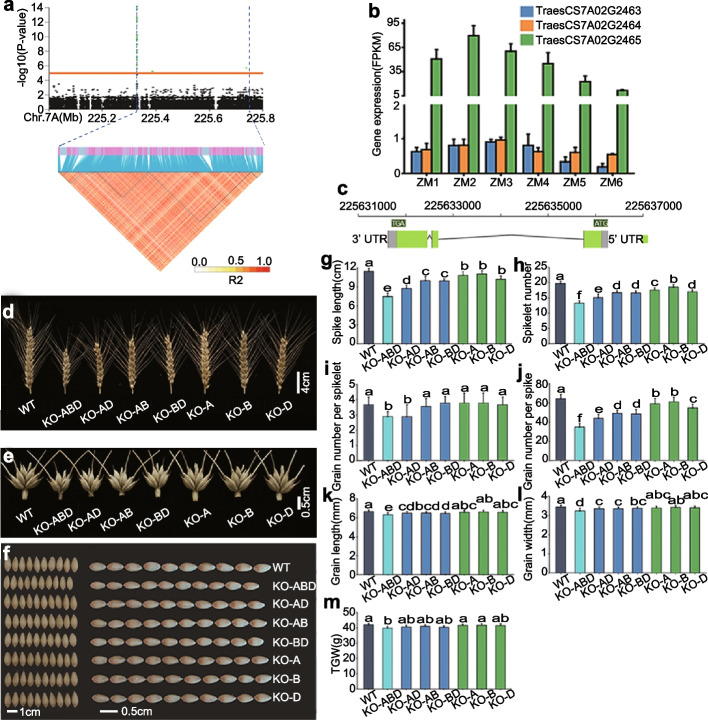
*TaSPL17-A* (*TraesCS7A02G246500*) is associated with spike morphology traits.** a** Local Manhattan plot and pairwise LD analysis showing the association between 20 markers and spike morphology trait in the region between 225,328,984 and 225,737,586 bp on chromosome 7A. Green dots indicate SNPs above the significance threshold (-log_10_[*P* value] = 5.00). Blue lines in the heatmap highlight strong LD with the significant variants. **b** Relative transcript levels for the three genes (*TraesCS7A02G246500*, *TraesCS7A02G246400*, *TraesCS7A02G246300*) within the associated region at six developmental stages: inflorescence meristem stage (ZM1), spikelet meristem stage (ZM2), glume primordium stage (ZM3), floral meristem stage (ZM4), stamen and pistil primordium stage (ZM5), and floral organ stage (ZM6). **c** Schematic diagram of *TraesCS7A02G246500*. Green rectangles and black lines indicate exons and introns, respectively. The gene is located on the reverse strand. **d**,** e** Spikes (**d**) and spikelets (**e**) of wild type (WT) and KO lines in the field. Scale bars = 4.00 cm (**d**) and 0.50 cm (**e**). **f** Grain width and length of WT and KO lines in the field. Scale bars = 1.00 cm and 5.00 mm for grain width and grain length, respectively. **g**–**m** Spike length (**g**), spikelet number per spike (**h**), grain number per spikelet (**i**), grain number per spike (**j**), grain width(**k**), grain length (**l**), and thousand grain weight (TGW) (**m**) in WT and KO lines in the field. Data are shown as means ± SD (*n* = 20). Triple KO line, KO-ABD; double KO line (*TraesCS7A02G246500* and *TraesCS7D02G245200* KO line), KO-AD; double KO line (*TraesCS7A02G246500* and *TraesCS7B02G144900* KO line), KO-AB; double KO line (*TraesCS7B02G144900* and *TraesCS7D02G245200* KO line), KO-BD; single KO line (*TraesCS7A02G246500* KO line), KO-A; single KO line (*TraesCS7B02G144900* KO line), KO-B; single KO line (*TraesCS7D02G245200* KO line), KO-D. Different lowercase letters indicate significant differences (*P* < 0.05), as determined by ANOVA

To further validate the power of the assimilate partitioning traits for detecting significant associations in GWAS, we present GWAS data for eight traits related to assimilate partitioning between spike components. These eight traits magnify the existing variation among different wheat accessions, allowing us to observe strong signals associated with grain weight and number. We detected different peaks for the eight traits on different chromosomes (Additional file 2: Figure S5), including peaks for awn weight. *OsSPL14* (*SQUAMOSA PROMOTER BINDING PROTEIN-LIKE14*, also named as *IDEAL PLANT ARCHITECTURE1* [*IPA1*]) in rice (*Oryza sativa* L.) is involved in the regulation of spike architecture, spikelet number, and tillering [33–36]. *OsSPL14* is a new Green Revolution gene that plays an important role in regulating plant architecture in rice [37–39]. *HvSPL14* in barley (*Hordeum vulgare* L.) is associated with very similar spike phenotypes for its KO lines [40]. *TaSPL17* is orthologous to *OsSPL14* [41, 42]; however, its effects on wheat plant architecture and grain yield remain largely unknown. Using the threshold =  -log_10_ [*P* value]> 5.00 according to previous reports [12, 23, 43–45], one identified genomic region (225,328,984 bp to 225,737,586 bp) contained *TaSPL17-A* (*TraesCS7A02G246500*) and another two genes (*TraesCS7A02G246400*, *TraesCS7A02G246300*) (Fig. 3a, Additional file 2: Figure S5). We extracted the relative transcript levels of the three genes at six developmental stages according to inflorescence morphological features from a published transcriptome dataset of wheat spike [46, 47]. Using the threshold =  - log_10_[*P* value] > 7.74 and identifying genes in 500 kb upstream and downstream according to previous work [23], we found five genes in 500 kb upstream and downstream of the 7A: 225,328,984-225,329,125 (Additional file 1: Table S10). Based on the results of these two approaches, only one gene (*TraesCS7A02G246500*) was expressed at high levels during spikelet and floret initiation and development [46, 47], making it a high-confidence candidate, as the other two genes were almost not detectable during this phase (Fig. 3b). The distance between *TraesCS7A02G246500* and the lead SNP was less than 400 kb (Additional file 1: Table S11). The homoeologous genes of *TraesCS7A02G246500* (Fig. 3c) are *TraesCS7B02G144900* (*TaSPL17-B*, B genome) and *TraesCS7D02G245200* (*TaSPL17-B*, D genome).

### Phenotypic analyses of knockout lines

To explore the potential contribution of *TaSPL17-A*, *TaSPL17-B*, and *TaSPL17-D* to grain yield traits in wheat, we generated knockout (KO) lines in all homoeologs by designing one single guide RNA (sgRNA) targeting a conserved region within the third exon of all three genes (Additional file 1: Table S12, Additional file 2: Figure S6). We confirmed the transgenic status of these lines using PCR primers specific for each homoeolog to amplify fragments from both the wild-type (WT) and edited loci. Accordingly, we identified seven independent homozygous lines covering all mutant combinations (Additional file 2: Figure S6). A 1-bp deletion and a 1-bp insertion were most frequently identified at the target site. All mutations caused a shift in the reading frame and introduced premature translation termination codons (Additional file 1: Table S12, Additional file 2: Figure S6). The KO lines exhibited clear effects on plant architecture and the eight traits related to spike assimilate distribution (Additional file 1: Table S13, Additional file 2: Figures S7 and S8). Although these eight traits magnified the variation between the different wheat accessions, they may also enlarge the extent of variation between replicates for each accession, leading to the observed inconsistent effects on related traits (Additional file 1: Table S13). In addition, we also observed substantial differences in plant architecture and spike morphology traits between WT and KO lines (Additional file 1: Table S13). Plants from the triple KO line (KO-ABD) harboring a mutation in all three homoeologs were shorter and produced more tillers than WT, but the double and single KO lines did not show significant differences for these two traits relative to WT (Additional file 1: Table S13, Additional file 2: Figure S7).

We observed consistent negative effects on spike length and spikelet number per spike in single, double, and triple KO lines ranging from 3.79 to 34.76% and from 5.84 to 32.74%, respectively (Fig. 3d, h, i, Additional file 1: Table S13). The triple KO line (KO-ABD) and one double KO line (KO-AD) exhibited a significant decrease in grain number per spikelet by 21.21% (0.78, *P* < 0.01) and 21.21% (0.78, *P* < 0.05), respectively, compared to WT (Fig. 3e, j, Additional file 1: Table S13). The other double and single KO lines did not obviously influence grain number per spikelet. The smaller spikelet number per spike, alongside the lower or unchanged grain number per spikelet, led to a 4.97-45.77% (3.20-29.45) decrease in grain number per spike in all the KO lines (Fig. 3k, Additional file 1: Table S13). The triple and double KO lines also showed a significant decrease in grain width and length (*P* < 0.05), translating into slight reductions in TGW in these three KO lines (Fig. 3f, l–m, Additional file 1: Table S13). Notably, KO lines had decreased grain number and grain weight (Fig. 2j, m), but they showed higher grain number/spike chaff and grain weight/chaff (Additional file 2: Figure S8). KO lines negatively affected both grains and chaff. We speculate that the higher grain number/spike chaff and grain weight/chaff maybe due to different sensitivities of grains and spike chaff to the knockout of this gene. This is consistent with the finding that some signals affecting grain number/size and assimilate partitioning traits were detected by GWAS of assimilate partitioning traits, but these signals were not observed in the GWAS results for grain number and size traits.

### Phenotypic analyses of overexpression lines

**Fig. 4:**
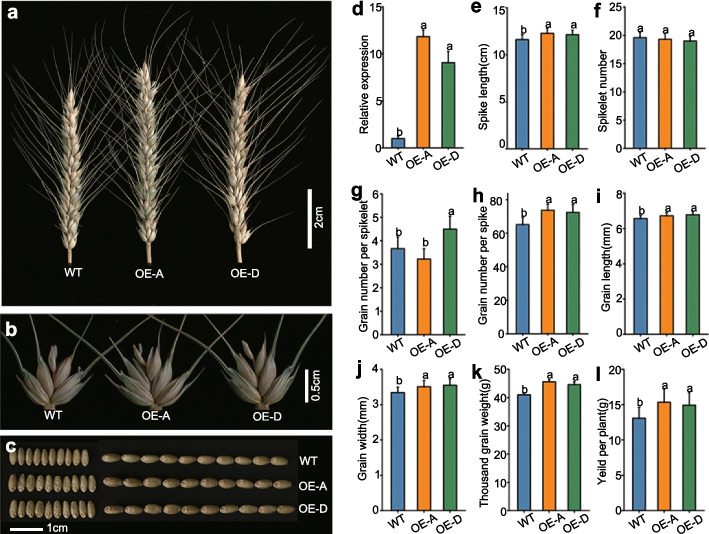
Phenotypic characterization of *TaSPL17 *OE lines in the field.** a**, **b** Spikes (**a**) and spikelets (**b**) of WT and OE plants. Scale bar, 2.00 or 0.50 cm for spike and spikelet, respectively. **c** Grain width and length of WT and OE lines. Scale bar = 1.00 cm. **d** Relative transcript levels of the corresponding genes in OE and WT. Values are means ± SD (*n* = 3). **e**–**l** Spike length (**e**), spikelet number per spike (**f**), grain number per spikelet (**g**), grain number per spike (**h**), grain length (**i**), grain width (**j**), TGW (**k**), and grain yield per spike (**l**) between WT and OE lines. Data are shown as means ± SD (*n* = 20). Different lowercase letters indicate significant differences (*P* < 0.05), as determined by ANOVA

To determine the effects of higher *TaSPL17* transcript levels, we generated transgenic overexpression lines (OE lines) by transforming WT wheat (Fielder cultivar) with a construct containing the full-length coding sequence of *TaSPL17-A* or *TaSPL17-D* driven by the maize (*Zea mays* L.)*Ubiquitin* promoter. The respective OE lines affected traits associated with assimilate partitioning between spike components (Additional file 2: Figure S8). In addition, we observed differences in plant architecture and spike morphology between WT and OE lines (Fig. 4a–l, Additional file 1: Table S14). We obtained two independent transgenic lines (OE-A for *TaSPL17-A*; OE-D for *TaSPL17-D*) with about tenfold higher transcript levels relative to WT (Fig. 4d, Additional file 1: Table S14). Plant height for the two OE lines was significantly shorter than that of WT (87.78 cm, WT; 79.42 cm, OE-A; 78.20 cm, OE-D; *P* < 0.05); however, the lines showed no obvious differences in tiller number (8.20, WT; 7.10, OE-A; 7.40, OE-D) (Additional file 1: Table S14). The two OE lines had increased spike length and grains per spike by 4.48-5.78% (0.52-0.67 cm) and 11.21-13.21% (7.30-8.60), respectively (Fig. 4e, h, Additional file 1: Table S14). Although we observed no significant difference between spikelet number per spike and grain number per spikelet (central spikelet) between OE-A and WT, apical and basal spikelets did have more grains in OE-A, which may explain the increased grain number per spike. The higher values measured for grain width and length resulted in an increase in TGW of 8.81-11.27% (3.61-4.62 g) (Fig. 4i–k, Additional file 1: Table S14) in the two OE lines relative to WT. The higher grain number per spike and higher TGW together with the unchanged tiller number led to a 14.05-17.23% (1.84-2.26 g) increase in grain weight per plant in the two OE lines (Fig. 4m, Additional file 1: Table S14). In summary, overexpressing *TaSPL17* increased grain yield per plant through its positive effects on grain number and size, highlighting the value of *TaSPL17* in wheat breeding.

### *TaSPL17* regulates the growth of the inflorescence meristem

The triple KO line in *TaSPL17* (KO-ABD) produced smaller spikes compared to WT starting at the spike initiation developmental stage throughout spike development with spikes in the triple KO line being less than half the size of WT spikes at the late stage (55 days after germination) (Additional file 2: Figure S9). The rachis expanded and spikelet primordia formed gradually in WT plants, whereas this developmental process was blocked in the triple KO line (Additional file 1: Table S13, Additional file 2: Figure S9). The rachis of the triple KO line was shorter and formed a small number of spikelet meristems before they converted to floret meristems, contributing to the formation of short spikes. The KO/OE lines also showed clear effects on the process of spike development. Compared to WT, the terminal spikelet stage (TS), yellow anther stage (YA), heading stage (HD), and anthesis stage (AN) were promoted in OE lines, but delayed in KO lines (Additional file 2: Figure S10). We concluded that overexpression of *TaSPL17* accelerates spikelet/floret initiation and development.

### *TaSPL17* expression pattern and subcellular localization of its gene product

**Fig. 5:**
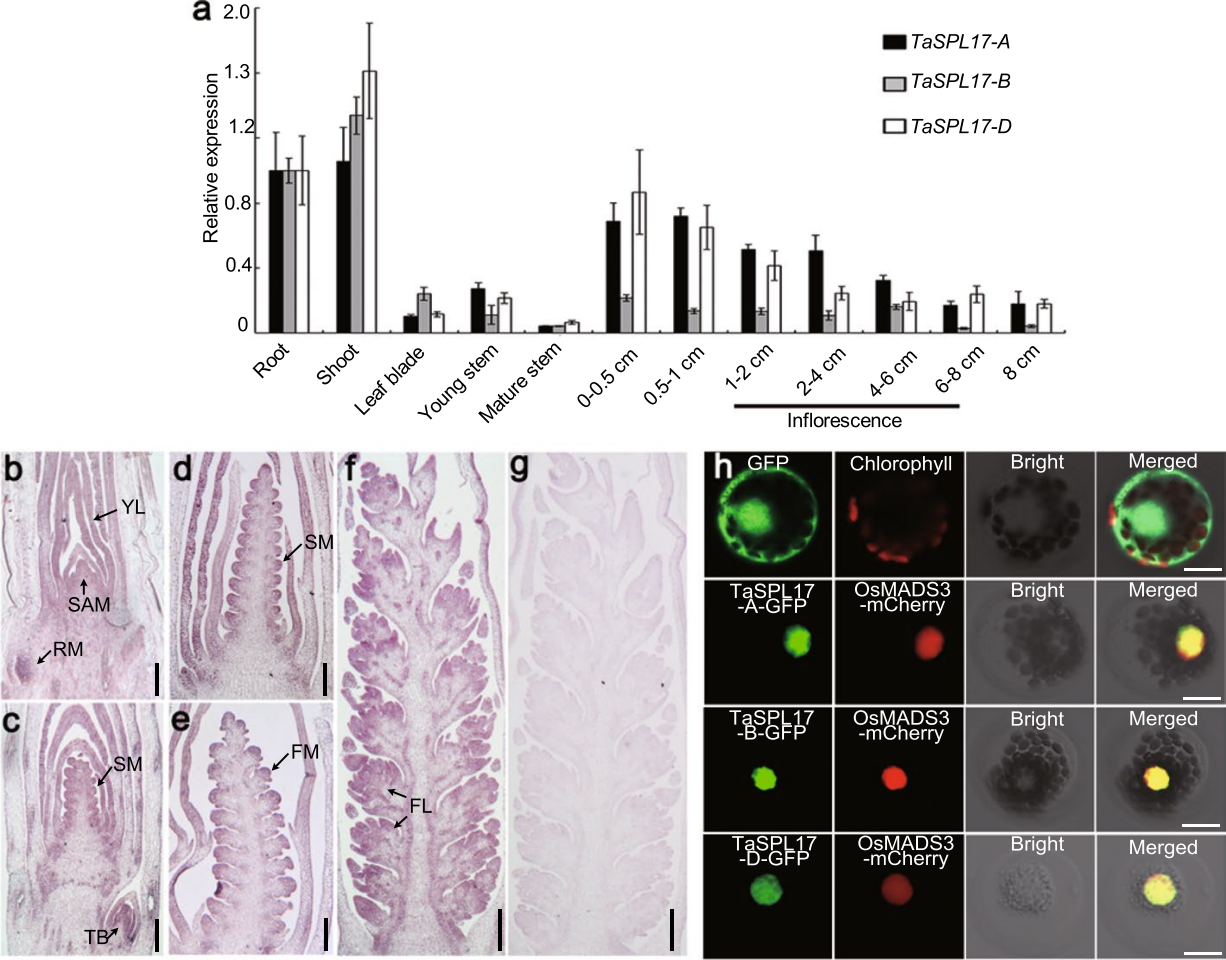
Expression pattern and subcellular localization ofTaSPL17.** a** RT-PCR analysis showing the relative transcript levels of *TaSPL17-A*, *TaSPL17-B*, and *TaSPL17-D* in various organs (root, shoot, leaf blade, young and mature stems, and inflorescence at different developmental stages). The wheat *Actin* gene was used as an internal control. Values are shown as means ± SD of three independent experiments and three biological replicates. **b**–**f** mRNA in situ hybridization reveals that *TaSPL17* is preferentially expressed in young leaves (YL), tiller buds (TB), root meristems (RM), the shoot apex meristem (SAM), spikelet meristems (SM), floret meristems (FM), and florets (FL). Arrows indicate the locations where *TaSPL17* is expressed. **g** *TaSPL17* sense probe was used as a negative control. **h** Subcellular localization of *TaSPL17* encoded by each subgenome. A construct encoding *TaSPL17-A*-*GFP*, *TaSPL17-B*-*GFP*, or *TaSPL17-D*-*GFP* was co-expressed with *OsMADS3-mCherry*, encoding a nuclear marker. From left to right: image of GFP (green), mCherry (red), protoplast, and merged GFP and mCherry. Scale bars, 200 μm in **b**-**e**, 500 μm in **f**, **g**, or 20 μm in **h**

We collected tissues from root, shoot, leaf blade, young stem, mature stem, and inflorescences at different developmental stages to measure expression levels of *TaSPL17-A*, *TaSPL17-B*, and *TaSPL17-D* (Fig. 5a). We detected high expression levels for all three homoeologs in roots and shoots, but low expression levels in leaf blades and mature stems. All three homoeologs were also highly expressed during early spike developmental stages, but their expression levels tended to decrease later with spike development. The expression level of *TaSPL17-B* was low relative to those of *TaSPL17-A* and *TaSPL17-D* (Fig. 5a). Considering its significant contribution to plant and spike morphogenesis, we examined the transcript pattern of *TaSPL17* in seedlings and young spikes at early differentiation stages using mRNA in situ hybridization. We determined that *TaSPL17* is strongly expressed in young actively dividing tissues such as root meristems, tiller buds, young leaves, and the shoot apical meristem. After the apex development entered the double ridge stage, *TaSPL17* was highly expressed in the spikelet meristem and floral primordia and remained highly expressed in the glume, stamen, and lodicule primordia during the development of floral organs (Fig. 5b-g).

To examine the subcellular localization of *TaSPL17*, we fused *TaSPL17* to the green fluorescent protein (GFP) and co-transfected the resulting encoding constructs with a nuclear marker into wheat leaf protoplasts. TaSPL17-A-GFP, TaSPL17-B-GFP, and TaSPL17-D-GFP fusion proteins all localized exclusively to the nucleus of wheat protoplasts (Fig. 5h), indicating that TaSPL17 is a nuclear protein and functions in the nucleus.

### Geographic differentiation and breeding selection of *TaSPL17* haplotypes

**Fig. 6:**
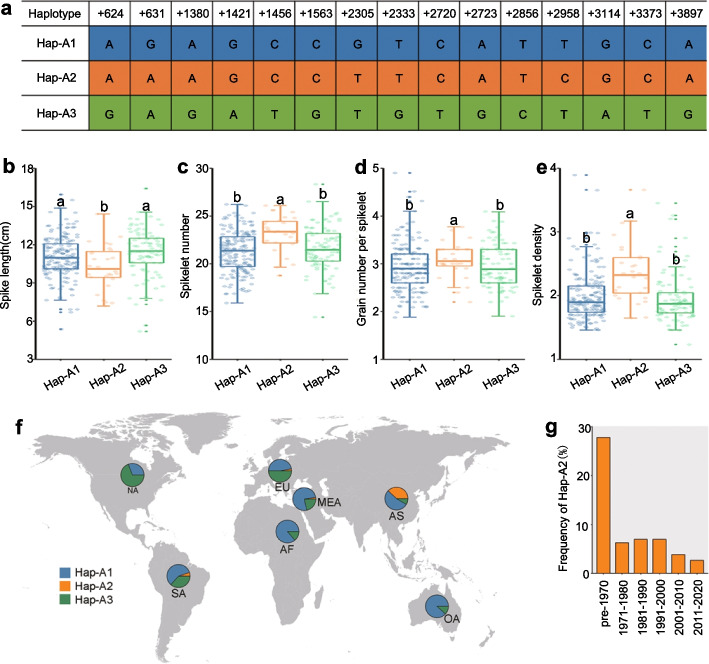
Haplotypes at *TaSPL17-A* affect spike morphology traits.** a** The three major haplotypes at *TaSPL17-A*. The first row indicates positions and the other three rows list the nucleotides for each haplotype. The positions are relative to the ATG of *TaSPL17-A*. + 624 and + 3897 indicate the first and last SNP in the three haplotypes of this gene. **b**-**e** Spike length (**b**), spikelet number per spike (**c**), grain number per spikelet (**d**), and spikelet density (**e**) between accessions carrying each of the three haplotypes (*n* = 173 accessions [Hap-A1], 28 accessions [Hap-A2], 93 accessions [Hap-A3]) of *TaSPL17-A*. Different lowercase letters indicate significant differences (*P* < 0.05). **f** Geographical distribution of the three haplotypes for each area (pie chart) AF, Africa; AS, Asia (except Middle East); EU, Europe; MEA, Middle East; NA, North America; OA, Oceania; SA, South America. **g** Frequency of Hap-A2 among 799 Chinese wheat accessions released from 1900 to 2020

Wheat breeding and domestication have exploited natural allelic variation that determines genomic regions and QTLs associated with agricultural traits. Understanding the genomic basis of this phenotypic variation in various germplasms is critical for making accurate selection decisions and for combining desired allelic combinations to improve wheat grain yield. We explored the genetic architecture of *TaSPL17-A* across the 306 worldwide wheat accessions used in this study, which revealed three major *TaSPL17-A* haplotypes that account for 93.79% (294 accessions) of all haplotypes (Fig. 6a, Additional file 1: Table S15). Haplotype-A1 (Hap-A1), haplotype-A2 (Hap-A2), and haplotype-A3 (Hap-A3) were present in 173, 28, and 93 accessions, respectively (Additional file 1: Table S15). We determined the effects of the three haplotypes on the associated traits (Fig. 6b-e, Additional file 1: Table S15). Compared to Hap-A1, the wheat accessions harboring Hap-A2 had higher average value of spikelet number per spike by 8.85% (1.88) but lower average value of spike length by 7.30% (0.80 cm), leading to a higher spikelet density of 19.08%. Hap-A2 also had higher average value of grain number per spike by 13.78% (6.25) (Additional file 1: Table S16). Among the three haplotypes, we only detected one nonsynonymous SNP (at position + 3897, with the ATG defined as + 1–3 bp) that alters a proline (Hap-A1, Hap-A2) to a leucine residue (Hap-A3, with the changed amino acid at position + 200) (Fig. 6a). Considering the phenotypic differences among the three haplotypes, this nonsynonymous SNP was unlikely to be responsible for the observed differences for grain number and size traits between Hap-A2 and Hap-A1 or Hap-A3.

The three major haplotypes of *TaSPL17-A* displayed worldwide geographical differentiation. For each geographical area, we determined the distribution of the three haplotypes (Hap-A1, Hap-A2, Hap-A3) among the local accessions. Hap-A1 had the same sequence as the reference genome (Chinese Spring). We observed that more accessions harbored Hap-A1 (33.33-88.24%) or Hap-A3 (11.11-66.67%) than Hap-A2 (0-36.51%) (Fig. 6f, Additional file 1: Table S17, S18). Hap-A1 contributed to a large fraction of accessions in Oceania (88.24%), Africa (86.21%), and Middle East (76.19%) relative to other areas (Fig. 6f, Additional file 1; Tables S17 and S18). Hap-A2 was mainly present in landraces from China (82.14%) (Fig. 6f, Additional file 1: Tables S19 and S20). Hap-A3 was more widely detected among wheat accessions from North America (66.67%), Europe (50.67%), and South America (36.59%) relative to other areas (Fig. 6f, Additional file 1: Table S18).

We further explored the history of *TaSPL17-A* haplotypes in 799 Chinese wheat accessions released between 1900 and 2020 (Fig. 6g, Additional file 1: Tables S21 and S22). Hap-A1 is positively associated with TGW and increased in frequency from 51.48% of all accessions before 1970 to 68.75% (1971-1980), 75.00% (1981–1990), 68.70% (1991-2000), 67.69% (2001-2010), and 69.54% (2011-2020) (Additional file 1: Tables S21 and S22). Hap-A2 conferring high spikelet number and grain number per spikelet was mainly observed before 1970 and decreased sharply in frequency after 1971 among Chinese wheat accessions (Fig. 6g, Additional file 1: Tables S21 and S22), suggesting that Hap-A2 was selected against in the past 50 years. Tajima’s *D* value of 4.16 for the 799 Chinese wheat accessions using VCFtools [48] indicated that these groups were subject to balanced selection. Due to its positive effects on grain number, Hap-A2 therefore has great potential for increasing grain yield in Chinese wheat breeding programs.

To explore the possible explanations for the effects of *TaSPL17-A* haplotypes on grain yield traits, we examined the differences between these three haplotypes. As mentioned above, the nonsynonymous SNP at position + 3897 was unlikely to explain the phenotypic differences associated with variations among the three haplotypes of *TaSPL17-A. SPL* transcripts are often targeted by microRNAs (miR) from the miR156 family [49, 50]. Although *TaSPL17-A* carried a miR-binding site in its third exon (chr7A: + 4070-4091), no SNP mapped to this region among the three haplotypes. Brassinosteroids (BRs) are plant hormones that play important roles in the regulation of plant growth and development. BRI1 EMS SUPPRESSOR 1 (BES1), a transcription factor, accumulates in the nucleus in response to BRs, where it binds to the promoters of and activates BR target genes to specifically regulate BR-mediated gene expression [51, 52]. One SNP (A/G, chr7A: 225,637,350) in the promoter region within a BES1-binding site may explain the phenotypic differences between Hap-A2 and the other two haplotypes. The alleles of the SNP were linked to the three haplotypes. Hap-A2 was linked to a G, while Hap-A1 and Hap-A3 were linked to an A at this position, suggesting a potential role for BRs in regulating spikelet/floret initiation and development.

We determined the LD value (*r*^*2*^) between the three haplotypes and the lead SNPs (Additional file 1: Table S23). The lead SNP: 7A-225,387,666 was in LD with one haplotype SNP: 7A-225,633,304 (*r*^*2*^ = 0.77). In addition, we identified seven SNPs in *TaSPL17-A* based on Sanger sequencing, and three of which were the same as the SNPs in the haplotypes (Additional file 1: Table S23). We further compared these SNPs with the lead SNPs to investigate causal variations. The lead SNP: 7A-225,328,984 (position: Chr. 7A: 225,328,984, the position in *TaSPL17-A*: + 3897) was identified by Sanger sequencing and was included in the haplotypes (Additional file 1: Table S24). We speculated that SNP: 7A-225,328,984 plays a key role in determining phenotypic variations in this study.

For *TaSPL17-B* (*TraesCS7B02G144900*), we identified four haplotypes in the 306 worldwide wheat accessions that account for 96.08% (294 accessions) of all haplotypes. Haplotype-B1 (Hap-B1), haplotype-B2 (Hap-B2), haplotype-B3 (Hap-B3), and haplotype-B4 (Hap-B4) were present in 88, 59, 129, and 18 accessions, respectively (Additional file 1: Tables S25 and S26). Hap-B1 and Hap-B2 exhibited high similarity to the reference genome relative to Hap-B3 and Hap-B4. Of these four haplotypes, the wheat accessions harboring Hap-B4 had the highest average value of spikelet number per spike, and grain number per spikelet, resulting in the highest grain number per spike of all Hap-B haplotypes (Additional file 1: Tables S25 and S26). The four haplotypes of *TaSPL17-B* displayed worldwide geographical differentiation. Hap-B2 and Hap-B3 accounted for relatively high percentages (38.98 and 27.91%, respectively) in Europe (Additional file 1: Tables S25 and S26). Hap-B1 displayed its highest frequency in Asia (except Middle East) (43.18%) (Additional file 1: Tables S25 and S26). Hap-B4 was more widely distributed in South America (44.44%) relative to other areas (Additional file 1: Tables S25 and S26). We did not observe obvious differences for *TaSPL17-B* (*TraesCS7B02G144900*) haplotypes among the 799 wheat accessions released from 1900 to 2020. We detected only one SNP for *TaSPL17-D* (*TraesCS7D02G245200*), which did not show obvious effects on grain yield traits.

To investigate the effects of *TaSPL17-B* haplotypes on grain yield traits, we examined the differences between these four haplotypes. We did not detect any differences in the coding sequence or in the miR-binding site (chr7B + 3678-3701) between the haplotypes of *TaSPL17-B*. However, we identified three SNPs (chr7B: 187,775,793 bp, C/T; chr7B: 187,775,941 bp, C/G; chr7B: 187,776,637 bp, C/G) in the promoter region of *TaSPL17-B*, differentiating Hap-B4 (C·····C·····C) from Hap-B1, Hap-B2, and Hap-B3 (T·····G·····G). We concluded that the variation in the promoter region may contribute to the phenotypic differences of Hap-B4 with the other three haplotypes by regulating the expression of *TaSPL17-B.*

We also assessed the haplotype diversity for *TaSPL17* across the A and B genomes in the 306 wheat accessions. We detected seven *TaSPL17-A* and *TaSPL17-B* haplotype combinations. In most cases, the examined phenotypic traits displayed greater differences in combinations of haplotypes compared to the *TaSPL17-A* or *TaSPL17-B* haplotypes alone (Additional file 1: Table S15). We observed the most marked differences between the two combinations Hap-A3 + Hap-B3 and Hap-A2 + Hap-B1 (Additional file 1: Table S15). Notably, compared to Hap-A3 + Hap-B3, the combination Hap-A2 + Hap-B1 had increased grain number per spike, grain number per spikelet, and spikelet density by 13.35% (7.10), 7.83% (1.84), and 21.44% (0.51), respectively (Additional file 1: Table S16).

### Utilization of *TaSPL17* alleles for the improvement of grain yield traits

To relate the contributions of *TaSPL17* alleles to grain yield traits and evaluate the value of *TaSPL17* in wheat breeding, we introgressed the *TaSPL17* allele (A/G at + 4245 bp in the gene, + 1046 bp in the cDNA) conferring high spikelet number, grain number per spikelet, and grain size to improve the potential of grain yield.

Jingshuang16 is a wheat accession with good winter hardiness that was widely planted during the 1980s and 1990s in Northern China. However, its spikelet number, grain number per spikelet, and grain size are relatively low, making it a prime candidate to test the use of an allele that improves these traits. We sequenced the coding sequence of *TaSPL17-A* in multiple accessions and detected a nonsynonymous SNP (A/G at + 4245 bp in the gene, + 1046 bp in the cDNA) between Bainong64 (an elite wheat cultivar widely planted in China) and Jingshuang16. The nonsynonymous SNP altered one amino acid (+ 316 protein sequence, Glu/Asp) from aspartate (Bainong64) to glutamate (Jingshuang16).

**Fig. 7:**
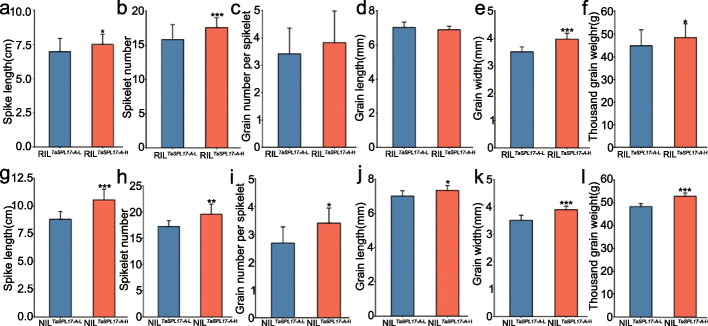
Introgression of *TaSPL17-A* favorable alleles in recombinant inbred lines (RILs) and near-isogenic lines (NILs) improves spike traits. Mean spike length, spikelet number, grain number per spikelet, grain width, grain length, and TGW are shown for original Jingshuang16 (RIL^*TaSPL17−A*−L^ first row [**a**-**f**], NIL^*TaSPL17−A*−L^ second row [**g**-**l**]) and improved Jingshuang16 (RIL^*TaSPL17−A*−H^ first row [**a**-**f**], NIL^*TaSPL17−A*^.^−H^ second row [**g**–**l**]). Data are shown as means ± SD. Significant differences were determined by Student’s *t* test (two sided, *P < 0.05, **P < 0.01, ***P < 0.001)

To understand the contribution of the detected SNPs on grain yield traits, we examined recombinant inbred lines (RILs) generated from the parents Bainong64 (RIL^*TaSPL17−A*−H^, carrying the *TaSPL17-A* allele conferring high spikelet number, grain length, grain width, and TGW) and Jingshuang16 (RIL^*TaSPL17−A*−L^, carrying the *TaSPL17-A* allele conferring low spikelet number, grain length, grain width, and TGW). On average, RIL^*TaSPL17−A*−H^ produced longer spikes, more spikelets per spike, wider and longer grains, and greater TGW than RIL^*TaSPL17−A*−L^ (Fig. 7, Additional file 2: Figure S11).

We also constructed a near-isogenic line (NIL) that carries the *TaSPL17-A*-H allele from Bainong64 through repeated backcrossing into Jingshuang16. Compared to the parental Jingshuang16 line carrying the *TaSPL17-A*-L allele (NIL^*TaSPL17−A*−L^), the improved Jingshuang16 carrying the *TaSPL17-A*-H allele (NIL^*TaSPL17−A*−H^) had longer spikes (8.80 cm for NIL^*TaSPL17−A*−L^, 10.60 cm for NIL^*TaSPL17−A*−H^, *P* < 0.001), more spikelets per spike (17.30 for NIL^*TaSPL17−A*−L^, 19.60 for NIL^*TaSPL17−A*−H^, *P* < 0.01) and more grains per spikelet (2.70 for NIL^*TaSPL17−A*−L^, 3.40 for NIL^*TaSPL17−A*−H^, *P* < 0.05), larger grains (grain width, 3.50 mm for NIL^*TaSPL17−A*−L^, 3.90 mm for NIL^*TaSPL17−A*−H^, *P* < 0.001), longer grains (7.00 mm for NIL^*TaSPL17−A*−L^, 7.40 mm for NIL^*TaSPL17−A*−H^, *P* < 0.05), and greater TGW (48.40 g for NIL^*TaSPL17−A*−L^, 53.00 g for NIL^*TaSPL17−A*−H^, *P* < 0.05) (Fig. 7, Additional file 2: Figure S11). These results suggest that *TaSPL17* alleles have the potential to improve wheat grain yield.

## Discussion

This study achieves two significant objectives: first, we developed a large-scale phenotype–genotype map of 27 spike morphology traits for 306 worldwide wheat accessions; second, we clarified the mechanism of *TaSPL17* regulating grain number and size in wheat. In addition, we elaborated on the geographical differentiation and breeding selection of *TaSPL17* haplotypes and their value in wheat breeding. For the first objective, we carried out GWAS using 40 million SNPs. Although a substantial number of wheat GWAS has been previously conducted, many of these studies on complex traits were underpowered owing to the relatively small number of SNPs used (< 1 million).

Several studies reported that LD for wheat genome was above 1 Mb (*r*^*2*^ = 0.50) [23, 43, 44]. In this study, based on *r*^*2*^ = 0.50, LD was 0.32, 0.007, 0.19 and 0.116 Mb for A, B, D, and whole genome, respectively. This observation is due to the strong genomic variation among the 306 worldwide wheat accessions. Traditionally, fine-mapping of a candidate gene in wheat would involve the development of several genetic populations and the identification of sufficient markers to obtain good genomic coverage, which may take several years. The large number of SNPs (~ 40 million SNPs, 2.8 SNPs per kb) and low LD used in this study allowed us to identify SNPs that are within or proximal to the candidate genes. This information may make it easier to obtain relatively precise locations of candidate genes.

Previous work identified very few major-effect genomic regions for grain yield, since grain yield is associated with grain number and size, which are influenced by different factors. We thus opted to dissect grain weight, number, and size using 27 traits. The GWAS for assimilate partitioning traits revealed strong signals that would not have been observed with GWAS conducted directly on grain yield, number, or size. The haplotypes at the identified peaks exhibited significant effects for the traits of grain number and size. The dissection of spike morphology using 27 traits allowed us to detect additional peaks, which is a powerful strategy to detect signals for grain yield traits in wheat.

Spikelet number per spike and grain number per spikelet are two determinants of grain number per spike in wheat. In addition, the “paired spikelet” is another promising trait for improving wheat spikelet number [53–56]. Spikelet number per spikelet is relatively stable in variable environments. Although a relatively large number of genes regulating spikelet number has been investigated [57–62], few studies have been reported in wheat on the molecular mechanism of floret fertility/grain number per spikelet [63], because grain number per spikelet exhibits high sensitivity in variable environments. In this study, we identified a gene regulating floret fertility/grain number per spikelet in wheat. The tradeoff between grain number and size negatively influences grain yield in wheat [64, 65]*.* We found that overexpressing *TaSPL17* increased grain number per spikelet and TGW, overcoming the tradeoff between these two determinants of grain yield in wheat.

This study also illustrated the connection between known genes and spike morphology traits. Our work suggests that *VRN-A1*, *VRN-A2*, *Ppd-D1*, and *TaFT-A5* are not only associated with spike length, spikelet number, and density, but also connected to the assimilate partitioning among spike components (Fig. 2, Additional file 1: Table S8). In addition, *TaAGL-A6*, *TaAGO1d-A*, *TaVRS-B3*, and *TaCEN-B* consistently displayed close connections with two traits exhibiting the assimilate distribution between grains and chaff within individual spikelet: grain weight/spikelet weight (individual spikelet), and chaff weight/spikelet weight (individual spikelet) (Fig. 2, Additional file 1: Table S8).

*TaSPL17* encodes a plant-specific transcription factor from the SPL family, which regulates the expression of target genes by binding to their cognate GTAC cis-element [66]. SPL transcription factors are closely associated with grain yield in crops. In rice, *OsSPL13* and *OsSPL14* promote the development of spike branches to enhance grain number per spike and grain yield [35, 37, 67]. *OsSPL16* determines grain size and shape and improves rice yield [68, 69]. Haplotypes of *TaSPL21* are associated with wheat TGW [70]. Ectopic expression of *TaSPL16* in Arabidopsis (*Arabidopsis thaliana*) increases organ size [71]. The indeterminate nature of wheat spikelets allows more than eight florets to form within one spikelet, in contrast to the fixed number in other cereals (rice, barley, and maize) [72–74]. Overexpressing *TaSPL13* increases florets and grains per spikelet in wheat [75]. By contrast, our results demonstrated that *TaSPL17* was highly expressed in spikelet and floral primordia and that the triple KO line lacking function of all three *TaSPL17* homoeologs suppressed the development of spikelets and florets, indicating that *TaSPL17* triggers spikelet and floret initiation and development.

Although some genes controlling grain number or size have been cloned, the effect of these genes in wheat breeding has been questioned. Our work explored the geographical distribution and breeding selection of *TaSPL17* haplotypes as well as their role in the improvement of grain yield in wheat. One haplotype of *TaSPL17* (Hap-A2), which improves grain number, was mainly present in landraces in China. We mainly identified Hap-A2 in Chinese wheat accessions released before 1970, and its frequency dropped from 1971 to 2020, suggesting that Hap-A2 was lost during modern wheat breeding in China. In China, wheat yield has achieved a great increase from the 1990s to today, mainly due to the genetic increase in TGW [76, 77]. Notably, the Yellow and Huai Winter Wheat Region is the most important wheat region in China. Wheat varieties in this region have relatively high spike number per unit area and medium grain number per spike. Although Hap-A2 exerts positive effects on grain number, it is not helpful in increasing grain size, which may partially explain why Hap-A2 was neglected during modern breeding in Chinese cultivars. When we introduced favorable *TaSPL17* alleles into modern wheat accessions, we observed improved grain number and size.

## Conclusion

In summary, our study provides a large amount of new genomic resources to further understand the genetic basis of spike morphology and to facilitate future breeding to improve wheat production.

## Methods

### Planting and phenotyping

For GWAS analyses, experiments were carried out in Zhaoxian, Hebei (37° 27′ N, 113° 30′ E, altitude 78 m) for two consecutive years. We planted six rows for each accession, and each row (1.50 m) included 15 plants, with 10.00 cm between rows. All field managements (e.g., irrigation, weed management, fertilization) were performed according to normal standards. Plants were irrigated when required. We measured 27 spike morphology traits at physiology maturity and calculated 15 ratios based on the 12 traits using 306 worldwide wheat accessions 2 years. We randomly selected the main shoots of five plants for each accession to measure the 12 traits. The 12 traits include spike length (cm), spike dry weight (g), grain weight per spike (g), awn weight per spike (g), spike chaff weight per spike (g), grain number per spike, total spikelet number per spike, rachis weight per spike (g), spikelet weight (g), grain number per spikelet, chaff weight per spikelet (g), grain weight per spikelet (g). Spike chaff includes glume, lemma, palea, and awn. Spike length does not include awns. Spikelet weight, grain number per spikelet, and chaff weight per spikelet were determined based on the spikelets in the center of spike.

Fruiting efficiency is often calculated at physiological maturity as the grain number per unit of chaff dry weight and has been proposed as a target trait to improve grain number and yield potential [78–80]. Therefore, fruiting efficiency was calculated for the entire spike and individual spikelets to use their results as traits for GWAS: grain number/spike chaff weight (entire spike) and grain number/spike chaff weight (individual spikelet). Wheat awns are long needle-like structures extending from the lemma in the florets and contribute to photosynthesis and grain production [81, 82]. Four traits were calculated to explore the contribution of awn to grains: awn weight/spike weight (entire spike), awn weight/grain weight (entire spike), awn weight/spike chaff weight (entire spike), and awn weight per spike. Wheat vascular bundles along the rachis constitute the transport system through which assimilates are transported to developing florets [83]. Grain weight/rachis weight (entire spike) was therefore used to assess the genetic connection between grains and rachis.

In total, the 12 measured traits were used to determine 15 traits to dissect spike components. The 15 traits comprised ten ratios for an entire spike and five ratios for individual spikelets. The ten ratios for the entire spike were thousand kernel weight ((grain weight/grain number)×1000), the ratio between grain number per spike and spike chaff per spike (grain number/spike chaff weight), the ratio between grain weight per spike and spike chaff weight (grain weight/spike chaff weight), the ratio between grain weight per spike and spike weight (grain weight/spike weight), the ratio between grain weight per spike and rachis weight per spike (grain weight/rachis weight), spikelet density (the ratio between total spikelet number and spike length, total spikelet number/spike length), the ratio between spike chaff weight and spike weight (spike chaff/spike weight), the ratio between awn weight per spike and spike weight (awn weight/spike weight), the ratio between awn weight per spike and grain weight per spike (awn weight/grain weight), and the ratio between awn weight per spike and spike chaff weight per spike (awn weight/spike chaff). The five ratios for individual spikelet traits consisted of the ratio between grain weight per spikelet and spikelet chaff (grain weight/spikelet chaff weight), thousand kernel weight ((grain weight/grain number) × 1000) for the spikelet in the center of spike, the ratio between grain number per spikelet and spike chaff weight (grain number/spike chaff weight), the ratio between grain weight per spikelet and spikelet weight (grain weight/spikelet weight), and the ratio between spikelet chaff weight and spikelet weight (spikelet chaff weight/spikelet weight).

For the phenotypic analyses of WT and transgenic lines, WT (cv. Fielder) and transgenic wheat were grown in an experimental field at the Chinese Academy of Agricultural Sciences in Beijing during the natural growing season. For the determination of grain width, length, spike length, and spikelet number per spike, the main shoots of 20 plants from each genotype were randomly selected. For the determination of grain number per spikelet (at the center of the spike) and TGW, nine and three spikes, respectively, were selected from the main shoots of different plants. For the RNA in situ hybridization and subcellular localization analysis, plants were grown in pots containing peat soil in controllable greenhouses with 70% relative humidity at 25 °C/20 °C, with a 16-h light/8-h dark photoperiod.

### Genotype calling and SNP identification

SNP discovery and genotyping used a workflow by Zhou Y for the construction of the whole-genome genetic variation map of wheat [20]. The latest version of VMap (VMap 2.0) is based on 1062 wheat accessions with multiple ploidy levels. We selected 306 hexaploid wheat accessions from the current version of VMap (VMap 2.0) for this study. A total of 40,710,923 segregating SNPs with MAF > 0.05 were used in the GWAS.

Genotype imputation estimates missing genotypes from the reference panel. Genotype imputation is commonly performed in GWAS because it greatly increases the number of markers [84–86]. The SNPs for 799 wheat accessions were obtained by genotype imputation based on the SNPs of 306 worldwide wheat accessions. Genotype imputations were conducted based on the source data according to previous work [84–86].

### Phylogenetic tree and population structure

To clarify the phylogenetic relationship from a genome-wide perspective, an individual-based neighbor-joining tree was constructed according to the *P* distance in Tassel (version 5.2.64) software and visualized using ITOL (https://itol.embl.de/). The population genetic structure was examined via an expectation maximization algorithm, as implemented in the program Admixture [87]. The number of assumed genetic clusters *K* ranged from 1 to 13, with 10,000 iterations for each run. Principle component analysis (PCA) was also performed to evaluate genetic structure in Plink (version 1.90b6.18) software. PCA was carried out to remove linked SNPs based on LD blocks and generated 2,756,291 SNPs for population structure analysis.

### LD analysis

To conduct pairwise LD analysis of the associated genomic region for the *TaSPL17-A,* the software PopLDdecay [88] was used to calculate the LD coefficient (*r*^2^) between pairwise high-quality SNPs; the parameters were set as follows: –allow-no-sex –maf 0.05 –geno 0.2 –r2 –ld-window 999999 –ld-window-r2 0.

To determine genomic intervals, the associated SNPs of all traits above a threshold of five were combined and de duplication were extracted by VCFtools software. LD between SNPs was calculated by Plink: plink –vcf chr_ position. vcf. recode. vcf –allow-no-sex –maf 0.05 –geno 0.2 –r2 –ld-window 50000 –ld-window-r2 0 –out out_ file_ prefix2. The results were used to combine SNPs to intervals. The significant markers were then delineated into intervals based on the LD between markers, where markers with *r*^*2*^ > 0.1 for the existence of LD were included in the same interval. If the distance between the peak SNPs of two adjacent loci was less than 5 Mb, these two loci were merged. The number of significant SNPs contained in the genomic region was also calculated. If the number of SNPs in corresponding genomic region was more than 10, these regions were defined as peaks or genomic regions. The most significant SNP means the peak SNP.

### Multiple comparisons and best linear unbiased estimates

The analysis was carried out using the agricolae package in R (agricolae: Statistical Procedures for Agricultural Research. R package version 1.3–1.https://CRAN.R-project.org/package=agricolae). The means of multiple groups were compared using the best significant difference (LSD) test, which required a one-way ANOVA.

The best linear unbiased estimates (BLUEs) for 27 traits across 2 years were calculated using the lme4 package in R [89] with the linear mixed linear model:

$$Y_{ij}\;=\;u\;+\;genoty_i\;+\;{\mathrm{Year}}_j\;+\;{\left(\mathrm{genoty}\;\times\mathrm{Year}\right)}_{ij}\;+\;{\mathrm{error}}_{ij}$$

Where *Y*_*ij*_ is the trait of the *i*th genotype in *j*th year; *u* is the mean; genoty_*i*_ is the genotype effect of the *i*th genotype; Year_*j*_ is the effect of the *j*th year; (genoty ×Year)_*ij*_ is the genotype-year interaction; error_*ij*_ is the error of the *j*th year and items were set to random.

### Broad sense heritability

Phenotypic data from all environments were analyzed by analysis of variance (ANOVA) in R. Broad sense heritability (H^2^) of 27 traits was calculated across environments from variance components according to the formula:

$$H2=\;\left(\sigma_G^2\right)/\left({\mathrm\sigma}_{\mathrm G}^2+\left(\sigma_{\mathrm{GE}}^2\right)/2+\left(\sigma_e^2\right)/2r\right)\;$$

Where ${\sigma }_{G}^{2}$ is the genotypic variance, ${\sigma }_{GE}^{2}$ is the genotype by the year effect, ${\sigma }_{e}^{2}$ is the residual error, 2 means two years and *r* is the average number of replications (*r* = 5).

#### GWAS

A large-scale GWAS on 27 spike morphology traits was carried out, using 40,710,923 SNPs (MAF > 0.05). Genome-wide association analysis performed in GEMMA (version 0.98.4) software using the mixed linear model (MLM) analysis:

$$y = X\alpha + S\beta + K\mu + e$$

where *y* represents phenotype; *α* and *β* are fixed effects representing marker effects and non-marker effects; and *μ* represents unknown random effects. *X*, *S*, and *K* are the incidence matrices for *α*, *β*, and *μ*, respectively, and *e* is a vector of random residual effects. The top three PCs were used to build up the S matrix in Plink (version 1.90b6.18). The matrix of simple matching coefficients was used to build up the kinship (K) matrix. The S matrix and kinship (K) matrix were performed for population structure correction. To determine the threshold, we performed permutation test for each trait by disrupting each 100 times and performed 100 GWAS analyses according to the MLM model. We ranked these *P*-values from smallest to largest for each trait and used a significance level of *α* = 0.5 and the 0.05 quantile of ranked *P*-values as the corrected significance level. The detailed results of the 100 runs of the traits are shown in Additional file 1: Table S27, with the significance levels of 7.67-18.93 for 27 traits. We also performed Bonferroni correction of 0.05/*n*, where *n* is the number of independent markers determined by Plink (version 1.90b6.18) (window size 50,000 bp, step size 5, *r*^2^ = 0.2, *n* = 2,756,291). This threshold of *P* value was determined by 0.05/2,756,291 (*P* = 1.81e - 07), - log_10_ [*P* value] = 7.74. To balance false positives and false negatives, so we conservatively chose - log_10_ [*P* value] = 5.00 as a moderate threshold for calling significant association according to previous GWAS work in wheat [12, 23, 43–45]. The GWAS results were visualized by Manhattan plots generated with the “CMplot” R package (https://github.com/YinLiLin/R-CMplot). The candidate genes in associated regions were identified and confirmed as significantly associated with the corresponding traits based on the two thresholds (- log_10_ [*P* value] = 5.0 and 7.74), which displayed consistent results. The trait-associated markers were aligned to the wheat reference genome RefSeq v.1.0 and visualized using Phenogram (http://visualization.ritchielab.org/phenograms/plot).

### RNA extraction and reverse transcription-quantitative PCR (RT-qPCR) analyses

Total RNA was extracted from various tissues using an RNeasy plant mini kit (Qiagen, Valencia, CA) in accordance with the manufacturer’s instructions and then treated with DNase I (TaKaRa, Tokyo, Japan) to remove genomic DNA contamination. First-strand cDNA was synthesized using a PrimeScript first-strand cDNA synthesis kit (TaKaRa, Tokyo, Japan). qPCR was performed using gene-specific primers and SYBR Premix Ex Taq reagents (Takara, Tokyo, Japan) on an ABI 7500 Fast real-time PCR system (Applied Biosystems, Foster City, CA). The wheat *Actin* gene was used as an internal control. qPCR was performed on three independent biological replicates. For each biological replicate, every sample was collected from at least six individual plants.

### Histological analysis

For microscopy observations, shoot apices of WT and the triple KO line (KO-ABD) plants were collected every 5 days from the vegetative stage, shortly before phase transition, to the end of floral differentiation. The shoot apices were carefully dissected and fixed in FAA solution (50% [v/v) ethanol, 5% [v/v) glacial acetic acid and 5% [v/v] formaldehyde). The samples were embedded in Paraplast (Leica Microsystems, Germany) after dehydration through a graded ethanol series (70% [v/v], 80%, 90%, and 100%) and hyalinized using a xylene:ethanol series with v/v ratios of 1:2, 1:1, 2:1, and 1:0. Then, the samples were sectioned into 8-μm sections using a Leica RM2245 rotary microtome. After removing Paraplast using a series of xylene and ethanol solutions, the sections were stained with toluidine blue before images were captured using a Leica ICC50 HD microscope.

### *RNA *in situ* hybridization*

RNA in situ hybridization was performed as described previously (Bradley et al., 1993) [90]. A specific 347-bp fragment of the *TaSPL17*-specific coding region was subcloned into the pGEM-T Easy vector (Promega, Madison, Wisconsin, USA) and used as a template to generate both antisense and sense RNA probes. Shoot apices of WT wheat seedlings at the third-leaf stage and young inflorescences were fixed using an RNase-free FAA fixative solution and then embedded in Paraplast (Leica Microsystems, Germany). Digoxigenin-labeled RNA probes were prepared using a DIG Northern starter kit (Roche) according to the manufacturer’s instructions. Slides were observed under brightfield using a Leica DMR microscope.

### Subcellular localization analysis

The coding sequences of *TaSPL17-A*, *TaSPL17-B*, and *TaSPL17-D* were individually cloned in-frame and downstream of the sequence of *GFP* and placed under the control of the cauliflower mosaic virus (CaMV) 35S promoter in the vector pAN580. Protoplasts were prepared from wheat mesophyll cells, and the *TaSPL17-A/B/D*-GFP fusion constructs and the nuclear marker *OsMADS3-mCherry* were co-transfected into wheat leaf protoplasts for transient expression using the polyethylene glycol (PEG4000)-mediated method. A Leica TCS-SP4 confocal microscope was used for fluorescence detection.

### Construction of gene editing and OE vectors

To generate mutants in *TaSPL17-A*, *TaSPL17-B*, and *TaSPL17-D (TraesCS7A02G246500*, *TraesCS7B02G144900*, and *TraesCS7D02G245200*, respectively), two sgRNA target sequences were designed according to the conserved exon sequences of *TaSPL17-A*, *TaSPL17-B*, and *TaSPL17-D* using the online tool CRISPR-P 2.0 (http://crispr.hzau.edu.cn/CRISPR2/). A segment containing the wheat *U3* promoter was amplified from the pCBC-MT1T2 vector using a pair of primers containing the two designed sgRNAs and then cloned into the BsaI site of the clustered regularly interspaced short palindromic repeats CRISPR/Cas9 vector pBUE411, using T4 DNA ligase (TransGen Biotech, China).

To simultaneously disrupt *TaSPL17* in all subgenomes (A, B, and D), two sgRNAs (sg1 and sg2) were designed targeting the conserved regions within the first and third exons of *TaSPL17-A*, *TaSPL17-B*, and *TaSPL17-D*, and the corresponding sgRNA/Cas9 vector was transformed into the wheat cultivar Fielder via Agrobacterium (EHA105) (*Agrobacterium tumefaciens*)-mediated transformation (Figure S3). Twenty bialaphos-resistant transgenic events harboring the T-DNA of the sgRNA/Cas9 vector were identified. *TaSPL17-A*, *TaSPL17-B,* and *TaSPL17-D*-specific PCR primers were designed to amplify fragments from both the WT *TaSPL17* and edited *TaSPL17* loci. DNA sequencing revealed that 50% (10 of 20) *T*_0_ events harbored compound heterozygous mutations at the gRNA2 site, whereas no mutations were detected at the gRNA1 site. Seeds of 10 mutant *T*_0_ events were collected and planted in the greenhouse, and 10 T_1_ plants for each *T*_0_ event were selected to detect mutation types at the gRNA2 site. A 1-bp deletion and a 1-bp insertion were most frequently identified at the target site. All mutations caused a frameshift leading to the introduction of premature translation termination codons.

To avoid the influence of potential off-target sites on phenotypic and functional analyses, the four most likely off-target sites with two or three mismatches were selected for amplification and analysis using specific primers. The results of sequencing did not reveal any mutations in the examined potential off-target sites of all 25 homozygous *TaSPL17* mutants.

To generate overexpressing plants, the coding sequences of *TaSPL17-A*, *TaSPL17-B*, and *TaSPL17-D* were individually amplified using DNA polymerase KOD FX (Toyobo, Osaka, Japan) from WT cDNA and cloned into the pMWB110 vector [91] using an In-Fusion Advantage PCR cloning kit (Clontech Laboratories, Takara, Tokyo, Japan). The resulting plasmids were transformed into the spring wheat cultivar Fielder via Agrobacterium-mediated transformation [92]. All primer sequences used in this study are listed in Supplemental Table S28.

### Supplementary Information

**Additional file 1: Table S1.** Summary of the 306 worldwide wheat accessions. **Table S2.** Diversity and genetic structure analyses of the 306 wheat accessions. **Table S3.** The determination of linkage disequilibrium (r2) in this study. **Table S4.** The heritability of 27 traits in this study. **Table S5.** The effects of genotype, environment, genotype ✖ environment interaction and heritability for the 27 traits in this study. **Table S6.** Overview of the 27 spike morphology traits in this study. **Table S7.** Detailed information of all significant associated SNPs for the investigated traits (-log10[P value] > 5.00). **Table S8.** Detailed information of all genomic intervals for the investigated traits. **Table S9.** The effects of 392 genomic intervals associated with assimilate partitioning for the grain yield traits. **Table S10.** The expression of candidate genes (FPKM). **Table S11.** The distance between lead SNPs and *TraesCS7A02G246500*. **Table S12.** Targeted mutagenesis of *TaSPL17* in different mutant lines. **Table S13.** The phenotypic data of knock out lines in this study. **Table S14.** The phenotypic data of overexpression lines in this study. **Table S15.** Overview of spike morphology traits among the haplotypes of *TaSPL17*. **Table S16.** The differences of spike morphology traits among the haplotypes of *TaSPL17*. **Table S17.** Worldwide distribution of the three major haplotypes of *TaSPL17-A*. **Table S18.** The percentage of worldwide distribution of the three major haplotypes of *TaSPL17-A*. **Table S19.** The Hap-A2 information of *TaSPL17-A* in 306 wheat accession. **Table S20.** The percentage of the three major haplotypes of *TaSPL17-A* in different regions. **Table S21.** The distribution of the twelve major haplotypes of *TaSPL17-A* in 799 Chinese wheat accessions. **Table S22.** The percentage of the three major haplotypes of *TaSPL17-A* in 799 Chinese wheat accessions. **Table S23.** The linkage disequilibrium (r2) between lead SNPs and haplotype SNPs. **Table S24.** The identified SNPs of *TaSPL17-A* by Sanger sequencing. **Table S25.** The distribution of the four major haplotypes of *TaSPL17-B* in different areas. **Table S26.** The distribution of the four major haplotypes of *TaSPL17-B* in different areas. **Table S27.** The results of permutation test. **Table S28.** Primers used in this study.

**Additional file 2: Figure S1.** Cross-validation (CV) errors of ADMIXTURE runs. **Figure S2.** Overview of phenotypic data according to the geographical origin of accessions. **Figure S3.** The distribution of phenotypic values of the 27 spike morphology traits for the 306 wheat accessions. **Figure S4.** The associations among the 27 spike morphology traits. **Figure S5.** Manhattan plot displaying the GWAS result of the eight traits. **Figure S6.** Homozygous targeted mutagenesis of *TaSPL17*. **Figure S7.** Phenotypic characterization of the *TaSPL17* mutant lines in field. **Figure S8.** Traits associated with spike assimilate partitioning in wild type (WT), overexpression (OE) and knock out (KO) lines. **Figure S9.** Early stages of spike development in wild type (WT) and the triple knockout (KO) line. **Figure S10.** Duration of the terminal spikelet stage (TS), yellow anther (YA), heading stage (HD) and anthesis stage (AN) in wild type (WT), the overexpression (OE) and the triple knockout (KO) lines. **Figure S11.** The spikes for NILs and RILs of *TaSPL17* in field.

**Additional file 3.** Review history.

## Data Availability

The genotypes of 306 wheat accession used in this study have been deposited in the Genome Variation Map (https://bigd.big.ac.cn/gvm) under accession number GVM000463 [20, 26–28]. The genes in Additional file 1: Table S8 [24, 30, 93–134] in the study were reported previously.
