# Supplementary material for: A high-resolution genotype–phenotype map identifies the TaSPL17 controlling grain number and size in wheat
Source: Genome Biol. 2023 Aug 28;24:196. doi: 10.1186/s13059-023-03044-2 (PMC10463835; doi:10.1186/s13059-023-03044-2)
Supplement: Supplementary file 2 — Additional file 2: Figure S1. Cross-validation (CV) errors of ADMIXTURE runs. Figure S2. Overview of phenotypic data according to the geographical origin of accessions. Figure S3. The distribution of phenotypic values of the 27 spike morphology traits for the 306 wheat accessions. Figure S4. The associations among the 27 spike morphology traits. Figure S5. Manhattan plot displaying the GWAS result of the eight traits. Figure S6. Homozygous targeted mutagenesis of TaSPL17. Figure S7. Phenotypic characterization of the TaSPL17 mutant lines in field. Figure S8. Traits associated with spike assimilate partitioning in wild type (WT), overexpression (OE) and knock out (KO) lines. Figure S9. Early stages of spike development in wild type (WT) and the triple knockout (KO) line. Figure S10. Duration of the terminal spikelet stage (TS), yellow anther (YA), heading stage (HD) and anthesis stage (AN) in wild type (WT), the overexpression (OE) and the triple knockout (KO) lines. Figure S11. The spikes for NILs and RILs of TaSPL17 in field. [file 13059_2023_3044_MOESM2_ESM.docx]

**
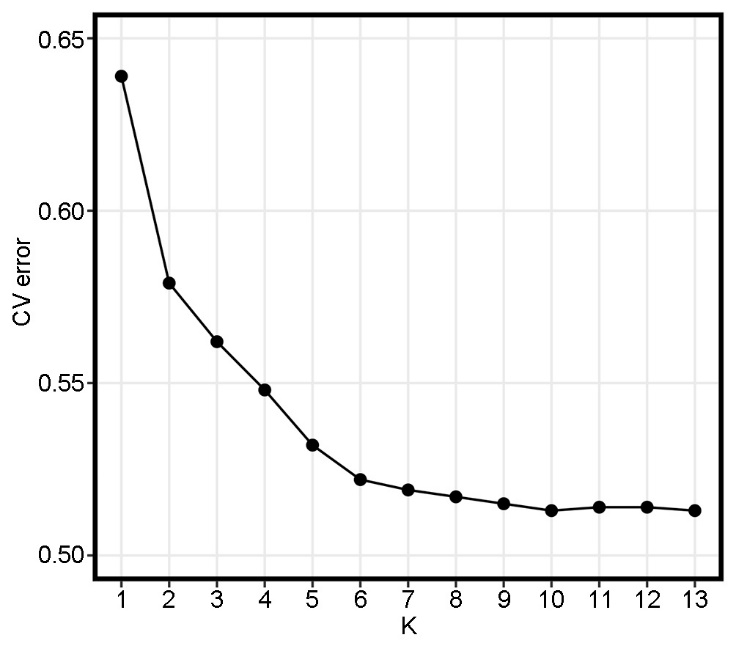
**

**Figure S1. Cross-validation (CV) errors of ADMIXTURE runs.** CV errors (y-axis) runs per K value (x-axis) are plotted.


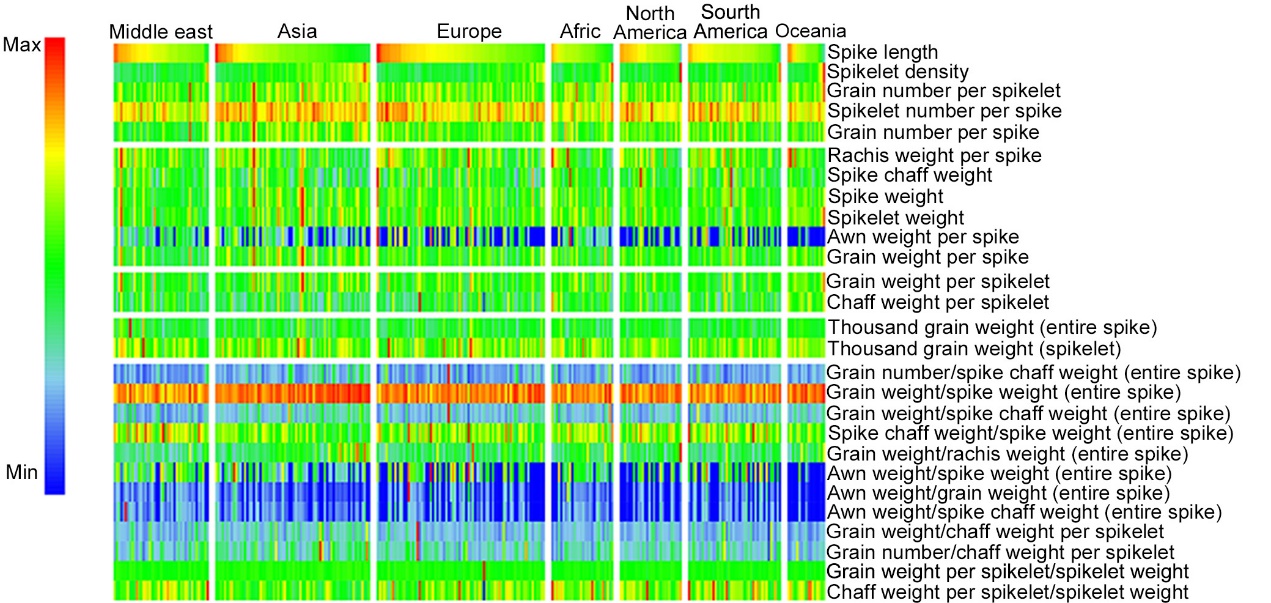


**Figure S2. Overview of phenotypic data according to the geographical origin of accessions.** The 27 traits are displayed for accessions sorted into seven geographical areas: Middle East (a part of Asia), Asia (except Middle East), Europe, Africa, North America, South America, and Oceania. The colors indicate variation of 27 traits among the 306 worldwide accessions. Entire spike means that the traits were determined based on the entire spike. The symbol “/” indicates the ratio between the two traits. All trait values from the heatmap are shown as the percentage of each value to the highest value.


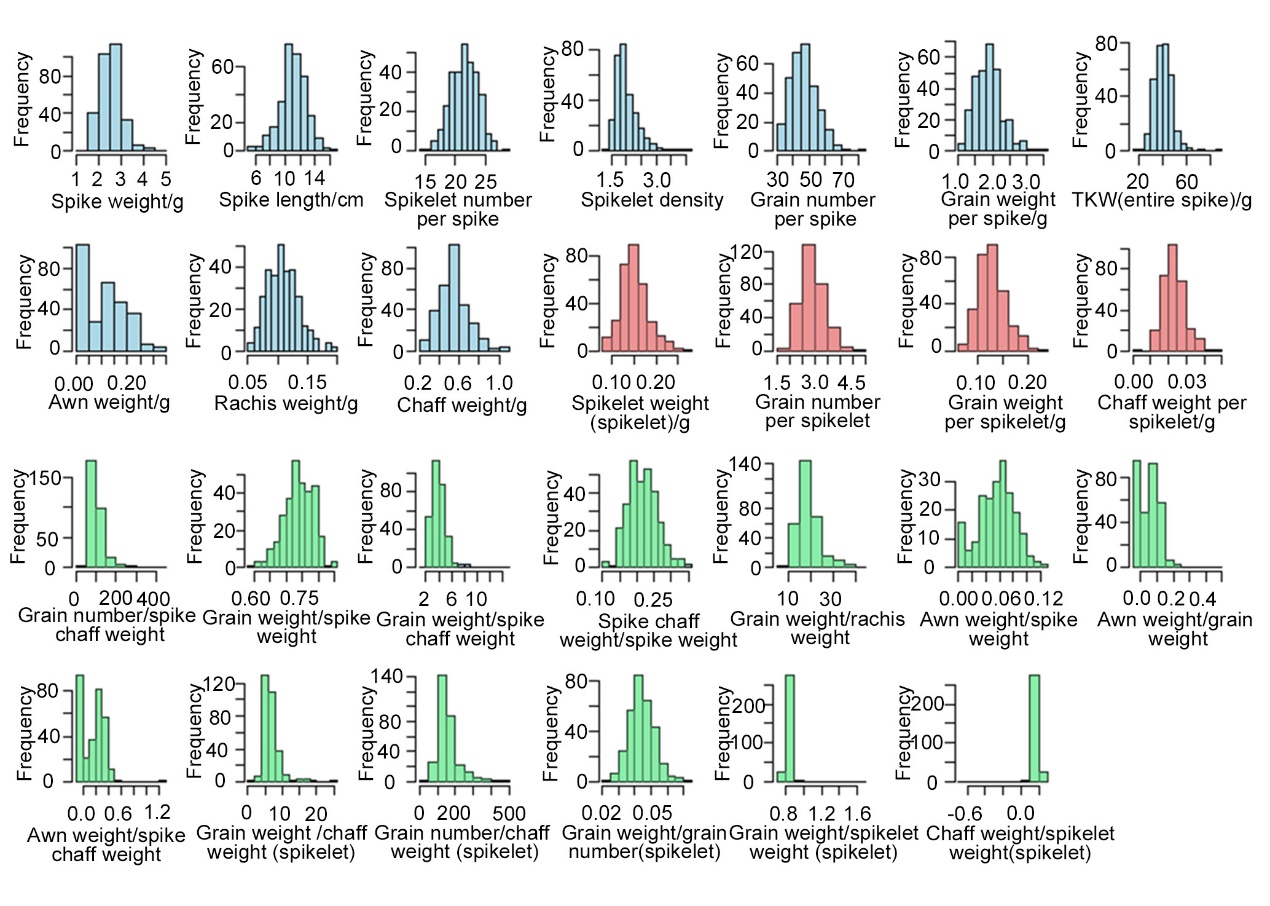


**Figure S3. The distribution of phenotypic values of the 27 spike morphology traits for the 306 wheat accessions.** The blue, red and green colors indicate the traits of the entire spike, the traits of individual spikelet, and the traits of ratios between spike components. The symbol “/” indicates the ratio between the two traits.


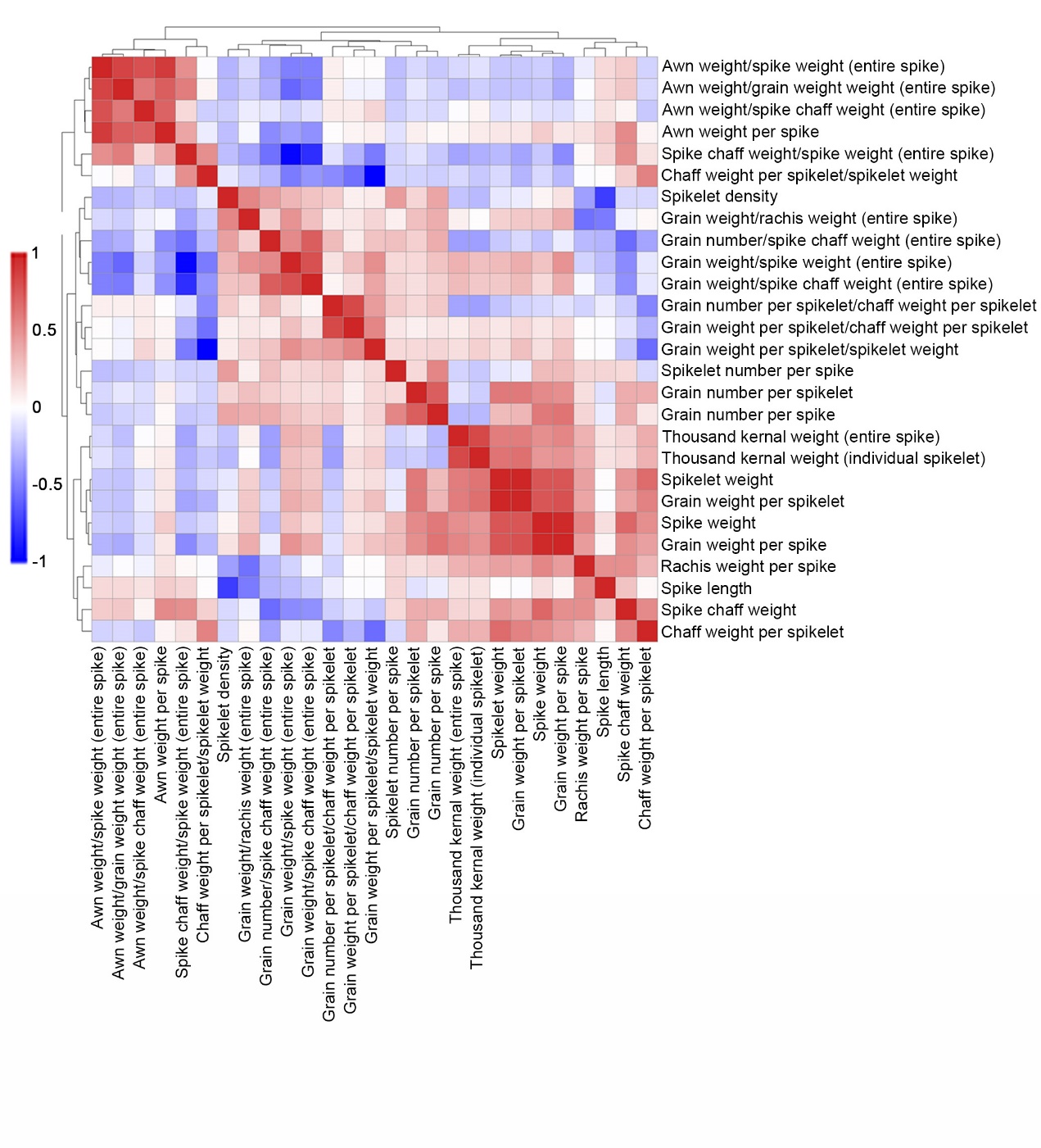


**Figure S4. The associations among the 27 spike morphology traits.** The associations were revealed by Pearson’s correlation coefficients, which were calculated using the phenotypic values for the 306 worldwide wheat accessions. The symbol “/” indicates the ratio between the two traits.


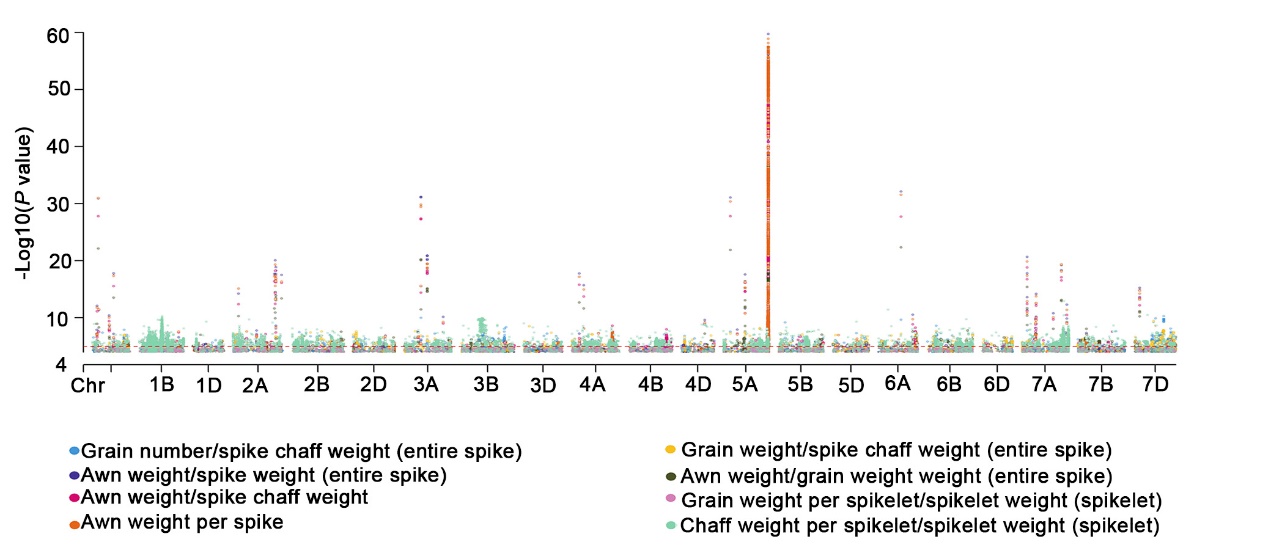


**Figure S5. Manhattan plot displaying the GWAS result of the eight traits.** The significantly associated SNP markers are labeled (-log_10_(P-value)>5.0). The eight traits suggest assimilate partitioning among spike components: grain number/spike chaff weight (entire spike), grain weight/spike chaff weight (entire spike), awn weight (entire spike), awn weight/spike weight (entire spike), awn weight/grain weight (entire spike), awn weight/spike chaff weight (entire spike), grain weight/spikelet weight (individual spikelet), chaff weight/spikelet weight (individual spikelet)

**
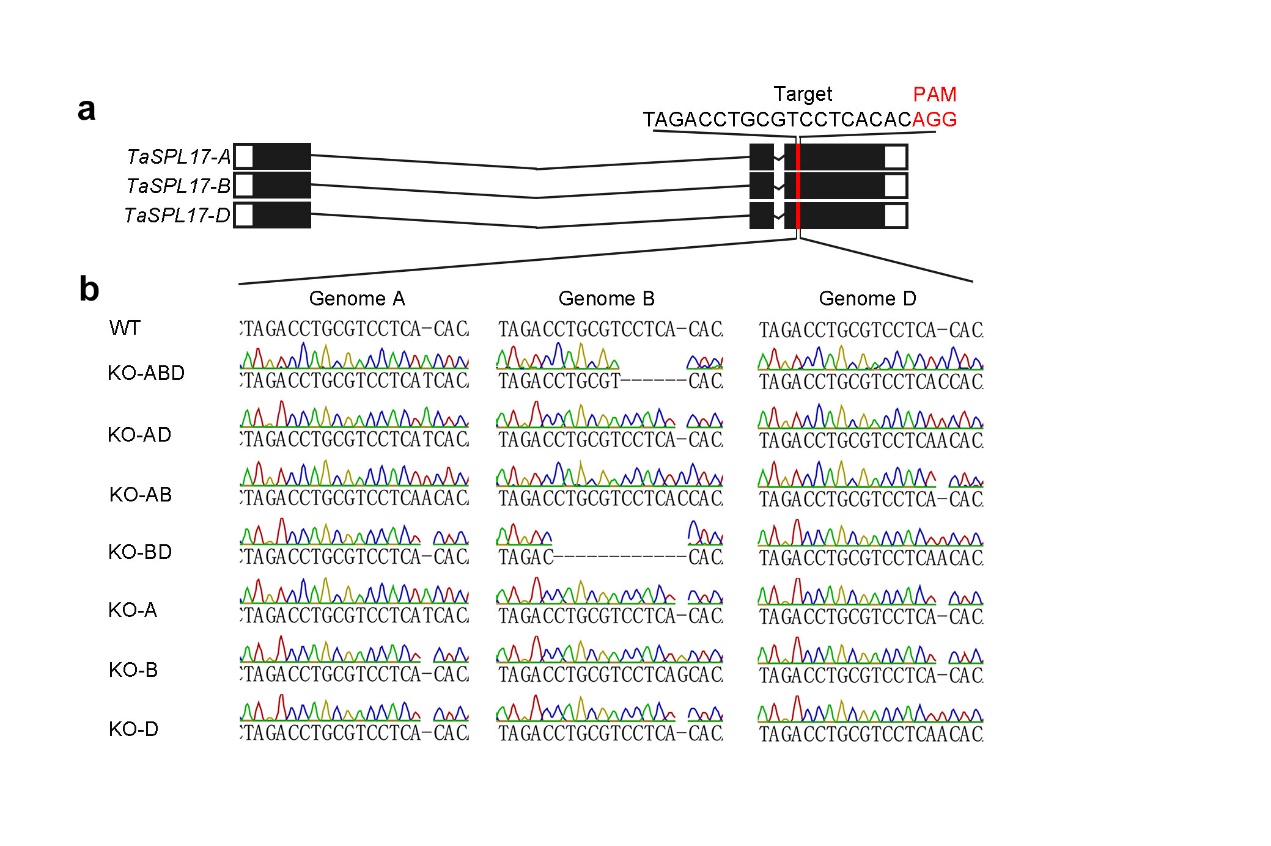
**

**Figure S6.** **Homozygous targeted mutagenesis of *TaSPL17*. a** Diagram of the single guide RNA target site in the conserved region of *TaSPL17-A*, *TaSPL17-B*, and *TaSPL17-D*. **b** Sequences and sequencing chromatograms corresponding to the wild type (WT) and seven representative edited individuals at target sites. “–” indicates the deletion of nucleotides.

**
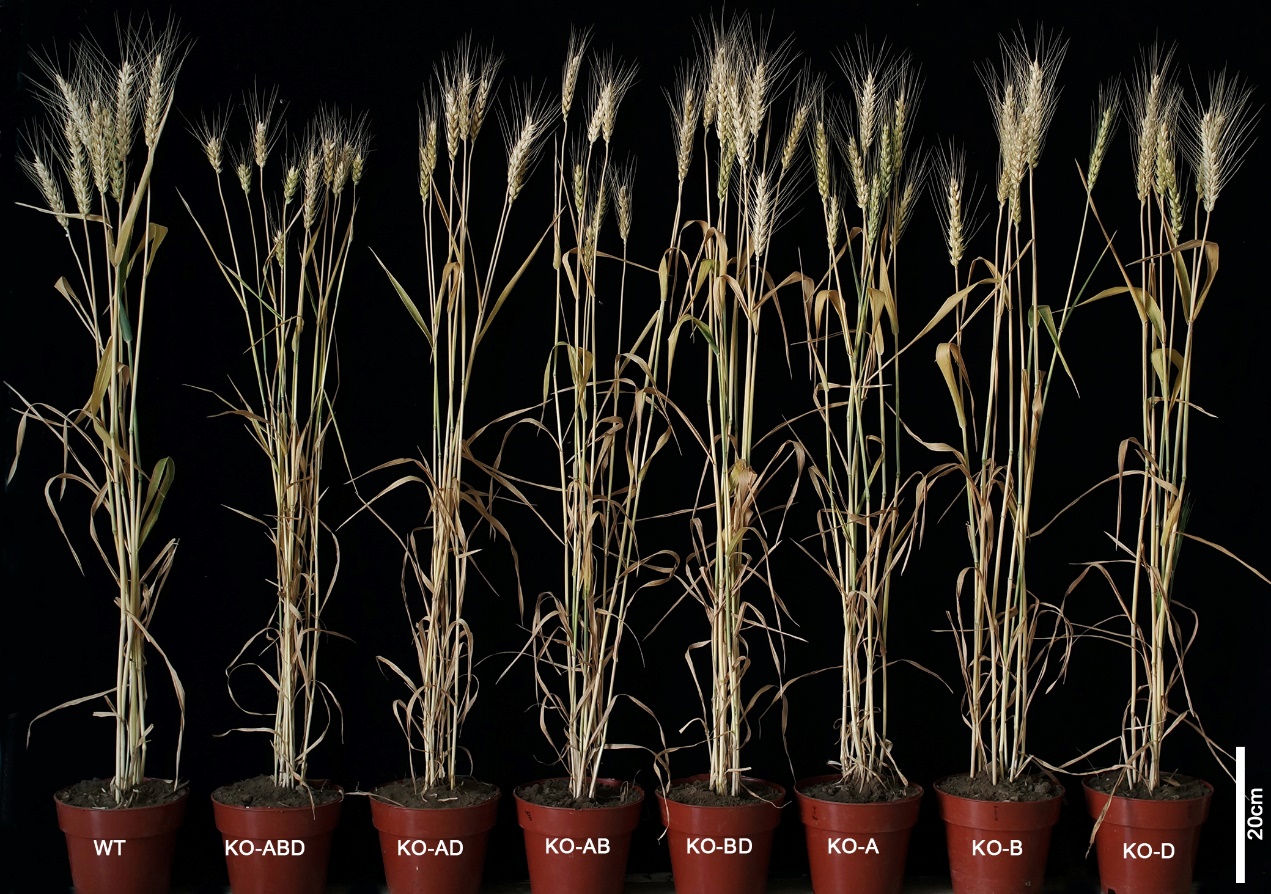
**

**Figure S7. Phenotypic characterization of the *TaSPL17* mutant lines in field.** Comparison of WT and *TaSPL17* mutant lines at the grain-filling stage. Scale bar = 20 cm.

**
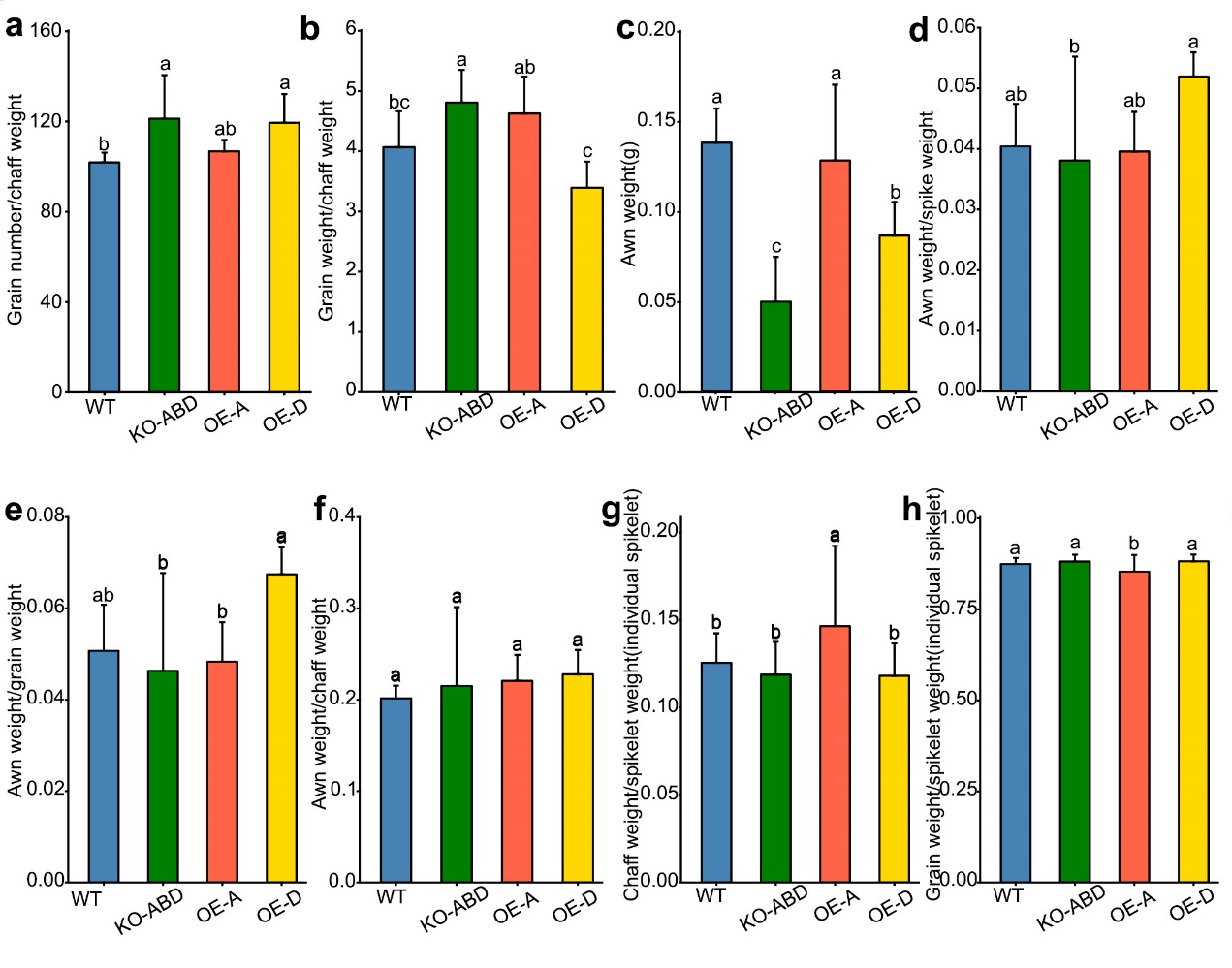
**

**Figure S8. Traits associated with spike assimilate partitioning in wild type (WT), overexpression (OE) and knock out (KO) lines.** **a**-**h** Comparison of eight traits that suggest assimilate partitioning among spike components: **a** grain number/spike chaff weight (entire spike), **b** grain weight/spike chaff weight (entire spike), **c** awn weight (entire spike), **d** awn weight/spike weight (entire spike), **e** awn weight/grain weight (entire spike) , **f** awn weight/spike chaff weight (entire spike), **g** chaff weight/spikelet weight (individual spikelet), **h** grain weight/spikelet weight (individual spikelet) between WT, OE-A, OE-D and triple KO line. Data are shown as means ± SD (n = 5). Different lowercase letters indicate significant differences (*P* < 0.05).


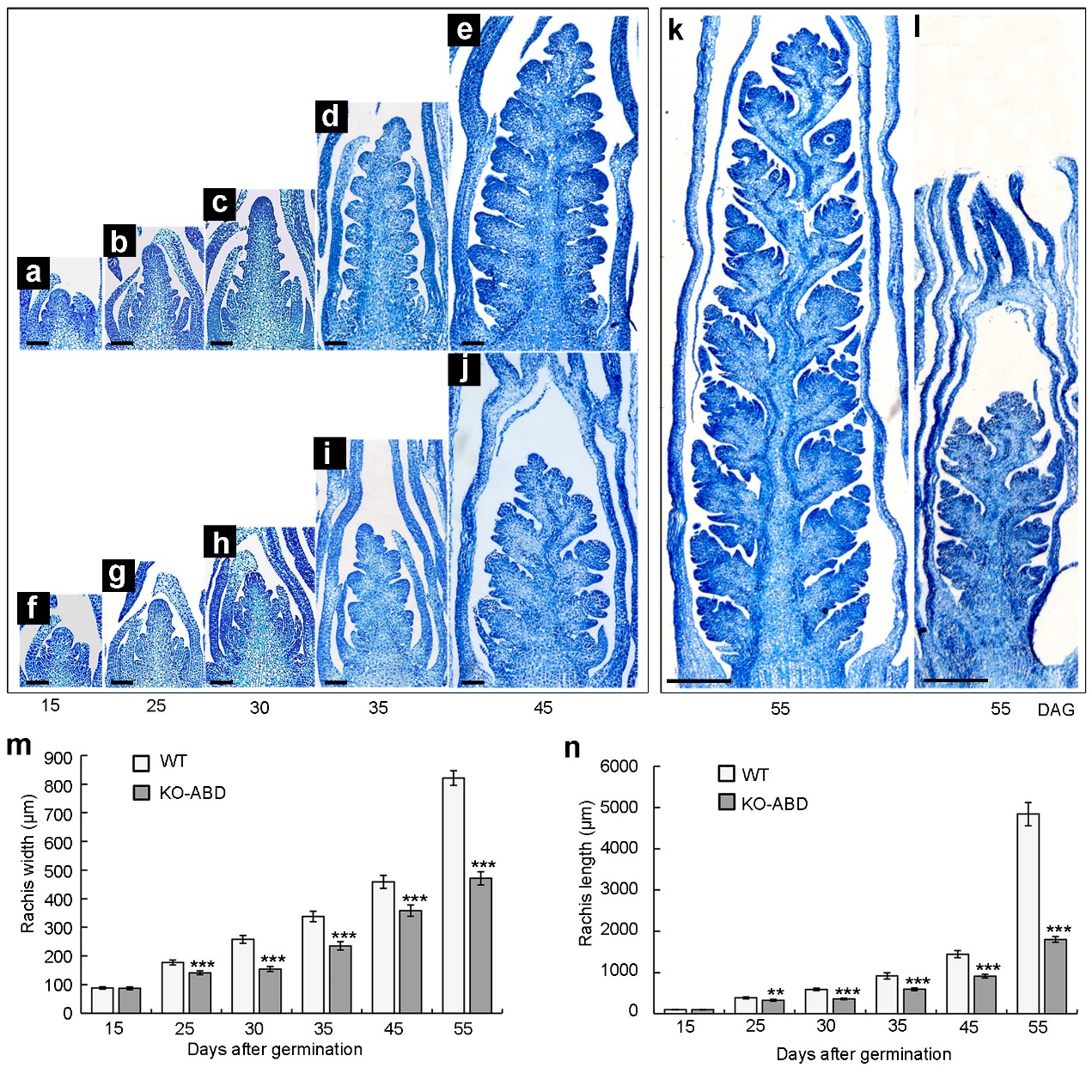


**Figure S9. Early stages of spike development in wild type (WT) and the triple knockout (KO) line. a–l** Histological analysis of inflorescence development in WT (**a–e** and **k**) and the triple KO line (**f–j** and **l**). Scale bars = 100 μm in (**a**–**j**) and 500 μm in (**k**, **l**). **m, n** Rachis width (**m**) and rachis length (**n**) in WT and the triple KO line. Data are shown as means ± SD (n = 6). Significant differences were determined by Student’s t-test (two sided, ***P* < 0.01, ****P* < 0.001).


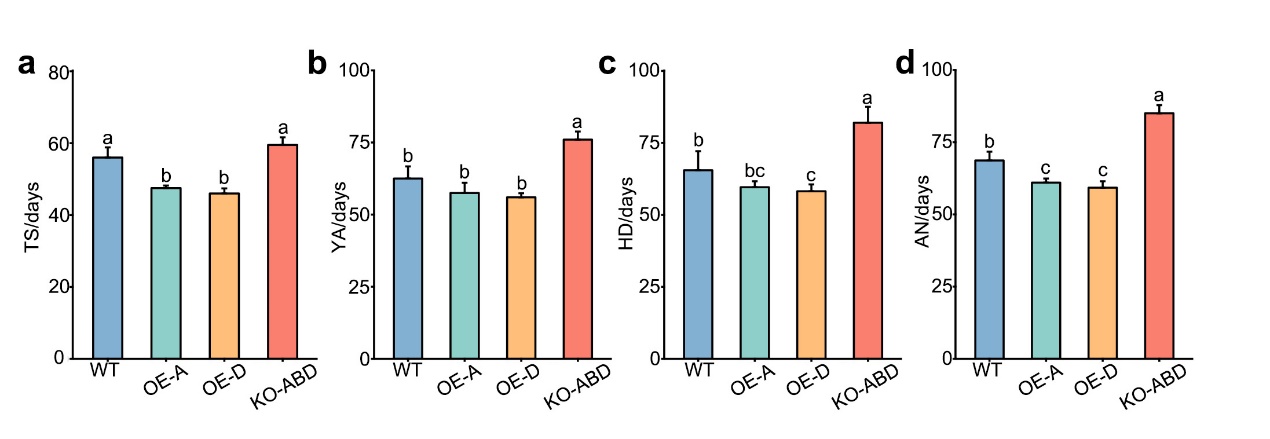


**Figure S10. Duration of the terminal spikelet stage (TS), yellow anther (YA), heading stage (HD) and anthesis stage (AN) in wild type (WT), the overexpression (OE) and the triple knockout (KO) lines(a-d).** Durations were determined from sowing by days. Data are shown as means ± SD (n = 5). Different lowercase letters indicate significant differences (*P* < 0.05).


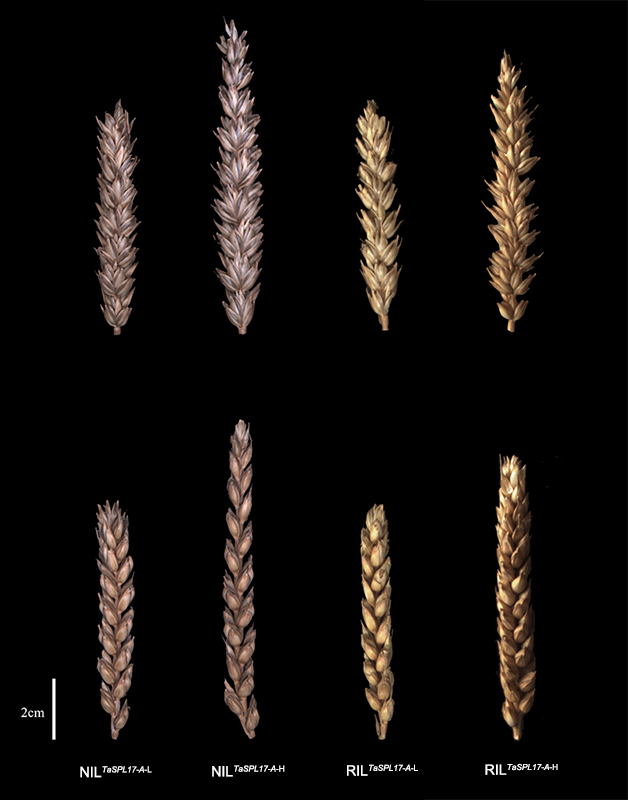


**Figure S11. The spikes for NILs and RILs of *TaSPL17* in field.** *TaSPL17-A*-L and *TaSPL17-A*-H indicate the lines with low (L) and high (H) alleles for the corresponding traits. The first and second rows show the front and side of the spikes for *TaSPL17-A*-L and *TaSPL17-A*-H.
