# Supplementary material for: A high-resolution genotype–phenotype map identifies the TaSPL17 controlling grain number and size in wheat
Source: Genome Biol. 2023 Aug 28;24:196. doi: 10.1186/s13059-023-03044-2 (PMC10463835; doi:10.1186/s13059-023-03044-2)
Supplement: Supplementary file 3 — Additional file 3. Review history. [file 13059_2023_3044_MOESM3_ESM.docx]

Review History

**First round of review**

**Reviewer 1**

**Were you able to assess all statistics in the manuscript, including the appropriateness of statistical tests used?**

Yes, and I have assessed the statistics in my report.

**Comments to author:**

In this manuscript, a total of 117,398,144 SNPs in the 306 wheat accessions were obtained through high-coverage whole-genome sequencing, the method of GWAS for 13 traits was used to show the associations. 453 genomic regions with strong signals have been identified to be associated with these traits. In addition, the authors identified a candidate gene HGY1 for spike morphology trait through gene expression analyses and transgenic assays. qRT-PCR and in situ hybridization analyses further confirmed the roles of this gene in spike development and inflorescence meristem growth. The authors also explored the genetic variations of HGY1 across the 306 wheat accessions, and constructed RILs and NILs with improved spike traits by introgression the elite allele of HGY1. Overall, this manuscript provides valuable information for the genetic improvement of grain yield-related traits in wheat.

However, several issues are also required for major revision:

1. The scientific writing has to be improved a lot. The abstract is too rough to get the precise and concise contribution of this study. The introduction section is not informative for readers to have solid background to realize these works. For the result section, results are mixed with many previous studies, which should be written in the introduction or discussion sections. Some parts are ambiguous. L206-208: Why did you focus on investigating the genomic region on chromosome 7A from totals of 453 genomic regions with strong GWAS signals. For the discussion section, you should spend more time to compare this study with the previous investigations, and focus on discussing the novel findings and precise contribution of this work, rather than just introduce numerous previous studies (such as L555-568).

2. More information of this work should be provided as figures or tables in the main text. For instance, it would be nice to provide manhattan plots for the GWAS results to directly show the distribution of all significant SNPs across the whole wheat genome. Because the plant architecture has significantly changed in HGY1 knockout and OE lines, it's better to provide some pictures for the whole plant of these transgenic lines and WT in the main text. In addition, the authors constructed RILs and NILs with improved spike traits by introgressing the elite allele of HGY1. It would be nice to provide the photos to show the differences of plant or spike morphology between RILs or NILs.

3. It was suggested that phenotypic variation is the basis of genetic mapping. Although the manuscript provided Supplementary Figure S2 to illustrate the variation of these 13 traits, more detailed analyses should be provided. Especially, the ANOVA table for these traits should be used to illustrate the variations of these traits among environments and genotypes. It is also nice to perform descriptive statistics for the 13 traits used in GWAS, and show the heritability of these traits as well as the correlation among them.

4. It is unclear how the authors detect candidate genes for significant SNPs. What are the criteria in defining the candidate region of Chr.7A? Which traits are associated with this region? How were the boundaries of this region determined? Does the interval of linkage disequilibrium decline contain only these three genes? More analyses details are required to evaluate the results. This is important considering that GWAS may have many false positives. Although the function of the candidate gene was verified, it is still necessary to define the criteria for determining candidate regions and picking candidate genes.

Specific issues:

Introduction:

L70: is critical for

L84-87: This paragraph should be integrated into the previous paragraph?

L88-92: Please provide more introduction for the significant novel findings of this work.

Results:

L139: Please provide the reference for the previous work in this sentence.

L146-148 and L152-171: There are too many introductions and explanations for these traits, which should be written into the sections of introduction or methods.

L178: I cannot find the information of these significant marker-trait associations across the three subgenomes in Figure 2A-D.

L199-200: It is more appropriate to discuss these contents in the discussion section.

Line 203: It's not clear what are the "eight detailed traits", and what is the purpose describing them in this paragraph. Are these traits used for the identification of the candidate region in Chr7? More detailed explanations are needed.

L206-208: As mentioned above, please provide more explanation for why you selected this genomic region on chromosome 7A from 253 regions with strong GWAS signals?

L295 and L310: Figure 3 include only A-L, and where is the Figure 3M?

L294-295: Please describe the differences in plant architecture and spike morphology between WT and OE lines more detail.

L298-299: Did you mean that plant height for the two OE lines was significantly shorter than that of WT?

L333-335: the terminal spikelet stage and anthesis stage were promoted in OE lines, but delayed in KO lines. We conclude that overexpression of HGY1 accelerate spikelet/floret initiation and development.

L391: between -> among

Lines 410-412: The changes of allelic frequencies are not sufficient for positive or negative selection of haplotypes. More careful population genetic analyses are needed to draw a valid conclusion. For example, Tajima's D or other tests.

Figure 6: It would be more convincing to provide photographs to show the phenotypes of the RILs and NILs.

L472: Please describe the difference between these two combinations more detailed.

Figure 5G: The axis labels are missing.

Discussion:

L529: The structure of discussion should be further improved to highlight the most significant contribution and novel findings of this work.

L555-568: As mentioned above, the authors just present the previous work in this paragraph. It would be nice to discuss and compared the roles of these genes in grain yield.

Supplementary Table S1: Latin names of species should be italicized.

**Reviewer 2**

**Were you able to assess all statistics in the manuscript, including the appropriateness of statistical tests used?**

Yes, and I have assessed the statistics in my report.

**Comments to author:**

This manuscript reported the GWAS analysis for traits related to assimilate distribution between grains and spike components. A candidate gene High Grain Yield1 (HGY1), encoding a SPL transcription factor was identified to positively control grain size and number by regulating the development of spikelet and floret meristems, resulting in enhanced grain weight per plant. The work about HGY1 function and utilization in wheat breeding is well planned and informative. However, the GWAS part does not make sense. My major concerns are:

1. The authors claimed that GWAS of assimilate partitioning between grains and spike chaff is a powerful strategy to detect signals for grain yield traits in wheat. I disagree with this conclusion due to a couple of concerns.

1) Lines 145-146: We observed strong signals in the GWAS results for these traits that cannot be revealed by GWAS conducted directly on grain yield, number or size. It is not reasonable and understandable that none of genomic locus was associated with grain yield, number or size. Also, it is meaningless for genomic regions related to dissected traits (13 traits in this MS) but not related to grain yield, number or size.

2) All of 13 traits are ratios of two agronomy traits, some of which indicated the assimilate distribution between grains and spike. Although these ratios were associated with grain yield, the ratio was not a good choice for GWAS analysis. Does the ratio normally distribute? KO lines showed decreased grain number and grain weight as shown in Figure 2, but they showed higher grain number/spike chaff and grain weight/chaff as shown in Supplemental Figure S4. Thus, ratio of grain number to spike chaff etc was not meaningful.

3) The genomic region (chromosome 7A: 225.33 Mb) was associated with 8 traits including Awn weight per spike, Awn weight/grain weight, Awn weight/spike chaff weight, Chaff weight per spikelet/spikelet weight, Grain number/spike chaff weight (entire spike), Grain weight per spikelet/spikelet weight, Grain weight per spikelet/spikelet weight? How to explain that the SPL TF affected awn weight?

4) TaSBEIIa, involved in starch biosynthesis, should be related to grain weight, but it was associated with grain number per spikelet/chaff weight per spikelet in this study (Supplemental Table S7). WAPO1 should be related to spikelet number per spike, but it is associated with grain weight/rachis weight (entire spike) in this study (Supplemental Table S7). The GWAS analysis in this MS is not supportive.

2. These 306 wheat accessions were mainly landraces collected from more than 70 countries. I wonder why the modern wheat cultivars showing elite agronomy traits were not included in this population.

3. Phenotypic Analyses of this population including phenotypic variation range, heritability, mean for all traits should be added in the results part and supplemental data. If the traits (ratios) were not normally distributed, GWAS was not available.

4. A total of 113,413 significant marker-trait associations were identified. Among such a huge scale of candidate loci, how to select this genomic region (chromosome 7A: 225.33 Mb) including only 20 marker-trait associations for further analysis?

5. How were the KO lines with single and double knocking out obtained, as three homoeologous genes were recognized by single guide RNA?

6. the results about HGY (SPL) affecting spike morphology and grain size were interesting and the evidence were solid. But the connection between GWAS and HGY functional analysis were weak.

Minor Concerns:

1) Wrong spelling for "homeologous". It should be homoeologous.

2) Line 38: encoding a transcription factor of the SQUAMOSA promoter-binding protein-like family. Family should be deleted.

3) Supplemental Figure S3: HGY1 was shown as TaSPL17, which will make the reader confused.

4) This MS was bad-written and should be improved by native English speaker.

**Reviewer 3**

**Were you able to assess all statistics in the manuscript, including the appropriateness of statistical tests used?**

Yes, and I have assessed the statistics in my report.

**Comments to author:**

This manuscript by Liu et al. provides an interesting advance in our understanding of the genetic control of yield related traits in wheat, and introduces us to the potential importance that a new gene in the form of an SPL transcription factor could play in improving the yield of wheat. In particular, the phenotyping approach used by the authors provide a new method to break down the complexity of yield as a trait, and the GWAS approach provides an excellent example of how new genomic sequence information could be used to uncover beneficial genetic variation in breeding programs. The paper included an excellent spread of molecular and genetic characterisation of this SPL transcription factor, and the potential yield improvements are considerable. The manuscript should have broad interest across plant sciences, breeding and biotechnology. I do have a few concerns which are outlined below.

MAJOR CONCERNS

L252-255: Can the authors please include the information in the main text about the gene edits that occurred in the transgenic lines, for each of the homeologs, and in doing so, provide more information about the nature of the "knock-outs"? For example, are they small deletions or insertions (if so, what size), are they missense mutations, deletions that cause frameshift or nonsense mutations? If they are small deletions and insertions, what is the consequence of these changes?

L277-278: To me, the gene nomenclature appears to be a contraction based on the name being derived from the KO lines, which clearly show reduced grain yield. Therefore, the gene name appears to refer to the wild-type, which is not high grain yield, but just relatively high compared to the knock-outs. I recommend the authors either better explain the basis of the gene nomenclature, or they change the gene name to reflect the phenotype of the knock-out lines, which follows convention.

L333-334: I don't agree with some of the conclusions made in this section, particularly that the KO lines have a delayed phase transition. For example, the KO line arrives at the TS stage at the same time as the WT. I agree that it flowers later, and therefore the time between TS and anthesis may be different, but without analysis of developmental stages in between TS and AN, I would be cautious about saying there is a phase transition phenotype. Also, did the OE-A line show a similar phenotype to OE-D for the data shown in Supp Figure 6?

L348: Regarding the in-situ hybridisation experiments, I would like to know if the authors can confidently say that the probe only bound to HGY-D1? The reason for asking this is that given probe sequences can be very similar between homeologs, I suspect that a HYG-D1 probe will also bind to HYG-A1 and HYG-B1. It may therefore be more accurate to state HYG-1 as the probe, rather than HYG-D1.

I have a few concerns regarding the haplotype analysis, especially regarding the assignment of causality. For example, 1) L385-388: based on what data are the authors concluding that Hap-A2 increases spikelet number, decreased spike length and increased grain number without having generated near-isogenic lines. These conclusions seem too strong based on the presented evidence. The same comment goes for L445-446 for HYG-B1. 2) In describing the haplotypes, it would help future studies if the authors made it clear which haplotype is most like the reference genome sequence. 3) In describing the haplotypes, it would help to understand if the phenotypes of these alleles, and the consequence of the sequence changes, fits with the data coming from the transgenic lines. For example, we would expect Hap-A2 has a gain-of-function affect to fit with the OE lines. Is this the case?

Following on from this, it is not clear which allele is used for the RILs and NILs from L488-527. For example, when the authors stage "we introgressed the HYG1 allele conferring high spikelet number...", what allele are they referring to and what haplotype/homeolog does this correspond to? In reference to the Bainong64 vs Jingshuang16 allele, what is the predicted functional consequence of this missense mutation? Is it in a domain of importance? Is it predicted to be deleterious? More information would help here.

The authors should include a section in the materials and methods explaining the statistical tests used in the paper.

MINOR CONCERNS

Line 219: correct to "TraesCS7A02G24600 are TraesCS7B02G144900" instead of "TraesCS7A02G24600 were TraesCS7B02G144900".

L298: Do the authors mean Fig. 3D here rather than 3E?

Figure 3: Are all of these images and data from plants grown in the glasshouse? Please include this detail in the figure legend if so.

L329: Why do authors refer to Supp Figure S4? Do they mean S5, here?

For all of the column graphs used in this article, I suggest the authors include the individual data point that contribute to the average and SEM.

In general, I found some of the results sections ended abruptly without summarising the outcome of a given set of experiments. For example, it would improve the readability to include summary sentences after L310, 357, etc.

L390: Please include the positional information for the amino acid change, e.g., PXXXL.

L559: When talking about FT2, the authors should cite Gauley and Boden, 2021, as they used hexaploid wheat whereas Shaw et al used tetraploid wheat.

**Authors Response**

**Point-by-point responses to the reviewers’ comments:**

Reviewer #1:

In this manuscript, a total of 117,398,144 SNPs in the 306 wheat accessions were obtained through high-coverage whole-genome sequencing, the method of GWAS for 13 traits was used to show the associations. 453 genomic regions with strong signals have been identified to be associated with these traits. In addition, the authors identified a candidate gene HGY1 for spike morphology trait through gene expression analyses and transgenic assays. qRT-PCR and in situ hybridization analyses further confirmed the roles of this gene in spike development and inflorescence meristem growth. The authors also explored the genetic variations of HGY1 across the 306 wheat accessions, and constructed RILs and NILs with improved spike traits by introgression the elite allele of HGY1. Overall, this manuscript provides valuable information for the genetic improvement of grain yield-related traits in wheat.

However, several issues are also required for major revision:

1. The scientific writing has to be improved a lot. The abstract is too rough to get the precise and concise contribution of this study. The introduction section is not informative for readers to have solid background to realize these works. For the result section, results are mixed with many previous studies, which should be written in the introduction or discussion sections. Some parts are ambiguous. L206-208: Why did you focus on investigating the genomic region on chromosome 7A from totals of 453 genomic regions with strong GWAS signals. For the discussion section, you should spend more time to compare this study with the previous investigations, and focus on discussing the novel findings and precise contribution of this work, rather than just introduce numerous previous studies (such as L555-568).

*Authors’ response 1: Thanks. According to the suggestions, we rewrote the abstract (line 30-68 in the revised manuscript with track changes), introduction (line 102-121 in the revised manuscript with track changes) and discussion (line 671-768 in the revised manuscript with track changes). We moved some parts of results to discussion (line 718-739 in the revised manuscript with track changes). Meanwhile, we provided more details for the selection of genomic region on chromosome 7A (line 309-333 in the revised manuscript with track changes). For the discussion section, we compared this study with the previous investigations and focus on discussing the novel findings and precise contribution of this work (line 684-742 in the revised manuscript with track changes). In addition, we sent the manuscript to language editing service. Please find the certificate below. We also included it in the attachment.*

2. More information of this work should be provided as figures or tables in the main text. For instance, it would be nice to provide manhattan plots for the GWAS results to directly show the distribution of all significant SNPs across the whole wheat genome. Because the plant architecture has significantly changed in HGY1 knockout and OE lines, it's better to provide some pictures for the whole plant of these transgenic lines and WT in the main text. In addition, the authors constructed RILs and NILs with improved spike traits by introgressing the elite allele of HGY1. It would be nice to provide the photos to show the differences of plant or spike morphology between RILs or NILs.

*Authors’ response 2: Thank you! According to the suggestions, we provided Manhattan plots for the GWAS results across the whole wheat genome (Additional file 2: Figure S5). In addition, we added the pictures for the whole plant of these transgenic lines and WT (Additional file 2: Figure S7) as well as the spike photos for RILs and NILs (Additional file 2: Figure S11).*

*Figure S5. Manhattan plot displaying the GWAS result of the eight traits. The significantly associated SNP markers are labeled (-log10(P-value)>5.0). The eight traits suggest assimilate partitioning among spike components: grain number/spike chaff weight (entire spike), grain weight/spike chaff weight (entire spike), awn weight (entire spike), awn weight/spike weight (entire spike), awn weight/grain weight (entire spike), awn weight/spike chaff weight (entire spike), grain weight/spikelet weight (individual spikelet), chaff weight/spikelet weight (individual spikelet).*

*Figure S7. Phenotypic characterization of the TaSPL17 mutant lines in field. Comparison of WT and TaSPL17 mutant lines at the grain-filling stage. Scale bar = 20 cm.*

*Figure S11. The spikes for NILs and RILs of TaSPL17 in field. TaSPL17-A-L and TaSPL17-A-H indicate the lines with low (L) and high (H) alleles for the corresponding traits. The first and second rows show the front and side of the spikes for TaSPL17-A-L and TaSPL17-A-H.*

It was suggested that phenotypic variation is the basis of genetic mapping. Although the manuscript provided Supplementary Figure S2 to illustrate the variation of these 13 traits, more detailed analyses should be provided. Especially, the ANOVA table for these traits should be used to illustrate the variations of these traits among environments and genotypes. It is also nice to perform descriptive statistics for the 13 traits used in GWAS and show the heritability of these traits as well as the correlation among them.

*Authors’ response 3: Thanks for the kind suggestions. We provided the distribution of phenotypic values to illustrate the variation of all the traits (line 181-183, 196-207 in the revised manuscript with track changes, Additional file 1: Table S4, Additional file 2: Figure S2, S3). In addition, we provided the ANOVA table for these traits to illustrate the variations of these traits among environments and genotypes (line 196-207 in the revised manuscript with track changes, Additional file 1: Table S4). We performed descriptive statistics for all traits used in GWAS (line 917-938 in the revised manuscript with track changes) and show the heritability of these traits as well as the correlation among them (line 208-215 in the revised manuscript with track changes).*

*The modifications in the manuscript (line 181-183 in the revised manuscript with track changes): “The 306 accessions displayed strong variation for all 27 traits (Additional file 1: Table S4, Additional file 2: Figure S2, S3).”*

*The modifications in the manuscript (line 196-207 in the revised manuscript with track changes): “The strong genetic effects of spike length, spikelet number per spike, awn weight per spike and spike density according to an analysis of variance (ANOVA) analysis were consistent with their relatively high heritability (Additional file 1: Table S4). The environmental effects and the influence of genotype × environment were significant for grain number and thousand-kernel weight (spikelet), which may explain their low heritability (Additional file 1: Table S4). The large residuals of spike chaff weight, chaff weight per spikelet, grain weight/chaff weight, grain weight per spikelet /chaff weight(spikelet), grain weight per spikelet /spikelet weight (spikelet) and chaff weight per spikelet /spikelet weight indicate that the dissection of these traits not only magnified the differences between genotypes, but also leaded to the strong variations among replicates within individual genotype, which resulted in low heritability (Additional file 1: Table S4).”*

*The modifications in the manuscript (line 208-215 in the revised manuscript with track changes): “The high-resolution dissection of spike morphology above captured several important relationships (Additional file 2: Figure S4). For instance, grain weight per spikelet/spikelet weight and chaff weight/spikelet weight were strongly and negatively correlated. Consistently, grain weight per spike/spike weight exhibited close and negative connections with spike chaff weight/spike weight, suggesting that there is strong competition for assimilates between spike chaff and grains. Awn weight per spike was strongly and positively associated with spike chaff weight, suggesting that awn is an important part of spike chaff.”*

*The modifications in the manuscript (line 917-938 in the revised manuscript with track changes):*

*Multiple comparisons and Best linear unbiased estimates*

*The analysis was carried out using the agricolae package in R (agricolae: Statistical Procedures for Agricultural Research. R package version 1.3-1.https://CRAN.R-project.org/package=agricolae). The means of multiple groups were compared using the best significant difference (LSD) test, which required a one-way ANOVA.*

*The best linear unbiased estimates (BLUEs) for 27 traits across two years were calculated using the lme4 package in R [86] with the linear mixed linear model:*

*Yij =u +genotyi +Yearj + (genoty *Year)ij + errorij*

*Where Yij is the trait of the i-th genotype in j-th year; u is the mean; genotyi is the genotype effect of the i-th genotype; Yearj, is the effect of the j-th year; (genoty *Year)ij is the genotype-year interaction; errorij is the error of the j-th year and items were set to random.*

*Broad sense heritability*

*Phenotypic data from all environments were analyzed by analysis of variance (ANOVA) in R. Broad sense heritability (H2) of 27 traits was calculated across environments from variance components according to the formula:*

*H2= (σG2)/(σG2+(σGE2)/2+(σe2)/2r)*

*Where σ_G^2 is the genotypic variance, σ_GE^2 is the genotype by the year effect, σ_e^2 is the residual error, and r is the average number of replications (r=5).*

*Figure S2. Overview of phenotypic data according to the geographical origin of accessions. The 27 traits are displayed for accessions sorted into seven geographical areas: Middle East (a part of Asia), Asia (except Middle East), Europe, Africa, North America, South America, and Oceania. The colors indicate variation of 27 traits among the 306 worldwide accessions. Entire spike means that the traits were determined based on the entire spike. The symbol “/” indicates the ratio between the two traits. All trait values from the heatmap are shown as the percentage of each value to the highest value.*

*Figure S3. The distribution of phenotypic values of the 27 spike morphology traits for the 306 wheat accessions. The blue, red and green colors indicate the traits of the entire spike, the traits of individual spikelet, and the traits of ratios between spike components. The symbol “/” indicates the ratio between the two traits.*

*Figure S4. The associations among the 27 spike morphology traits. The associations were revealed by Pearson’s correlation coefficients, which were calculated using the phenotypic values for the 306 worldwide wheat accessions. The symbol “/” indicates the ratio between the two traits.*

4. It is unclear how the authors detect candidate genes for significant SNPs. What are the criteria in defining the candidate region of Chr.7A? Which traits are associated with this region? How were the boundaries of this region determined? Does the interval of linkage disequilibrium decline contain only these three genes? More analyses details are required to evaluate the results. This is important considering that GWAS may have many false positives. Although the function of the candidate gene was verified, it is still necessary to define the criteria for determining candidate regions and picking candidate genes.

*Authors’ response 4: Thank you! According to the suggestions and comments, we provided more details for the selection of genomic region on chromosome7A (line 309-333 in the revised manuscript with track changes). The SNP marker-trait association within this region are shown in (Figure S5 and Table S6). Moreover, we clarified the boundaries of this region (line 904-915 in the revised manuscript with track changes). We compared the LD in this study with previous work to show that our work has significant narrowed down the LD in wheat (line 698-701 in the revised manuscript with track changes).*

*The modifications in the manuscript (line 309-333 in the revised manuscript with track changes): “To further validate the power of the assimilate partitioning traits for detecting significant associations in GWAS, we present GWAS data for eight traits related to assimilate partitioning between spike components. These eight traits magnify the existing variation among different wheat accessions, allowing us to observe strong signals associated with grain weight and number. We detected different peaks for the eight traits on different chromosomes (Additional file2: Figure S5), including peaks for awn weight. OsSPL14 in rice (Oryza sativa) (SQUAMOSA PROMOTER BINDING PROTEIN-LIKE14, also named as IDEAL PLANT ARCHITECTURE1 [IPA1]) is involved in the regulation of spike architecture, spikelet number and tillering [31-34]. OsSPL14 is a new "Green Revolution" gene that plays an important role in regulating plant architecture in rice [35-37]. HvSPL14 in barley (Hordeum vulgare) is associated with very similar spike phenotypes for its KO lines [38]. TaSPL17 is orthologous to OsSPL14 [39, 40], however, its effects on wheat plant architecture and grain yield remain largely unknown. One identified genomic region (225,328,984 bp to 225,737,586 bp) contained TaSPL17-A (TraesCS7A02G246500) and another two genes (TraesCS7A02G246400, TraesCS7A02G246300) (Fig 3a, Additional file2: Figure S5). We extracted the relative transcript levels of the three genes at six developmental stages according to inflorescence morphological features from a published transcriptome dataset of wheat spike [41, 42]. Only one gene (TraesCS7A02G246500) was expressed at high levels during spikelet and floret initiation and development, making it a high-confidence candidate, as the other two genes were almost not detectable during this phase (Fig. 3b).”*

*The modifications in the manuscript (line 698-701 in the revised manuscript with track changes): “Several studies reported that LD for wheat genome was above 1Mb (r2=0.5) [24, 48, 49]. In this study, based on r2= 0.5, LD was 0.32Mb, 0.007Mb, 0.19Mb and 0.116Mb for A, B, D and whole genome, respectively. This observation is due to the strong genomic variation among the 306 worldwide wheat accessions.”*

*The modifications in the manuscript (line 904-915 in the revised manuscript with track changes): “To determine genomic intervals, the associated SNPs of all traits above a threshold of five were combined and de duplication were extracted by VCFtools software. LD between SNPs was calculated by Plink: plink --vcf chr_ position. vcf. recode. vcf --allow-no-sex --maf 0.05 --geno 0.2 --r2 --ld-window 50000 --ld-window-r2 0 --out out_ file_ prefix2. The results were used to combine SNPs to intervals. The significant markers were then delineated into intervals based on the LD between markers, where markers with r2 > 0.1 for the existence of LD were included in the same interval. If the distance between the peak SNPs of two adjacent loci was was less than 5Mb, these two loci were merged. The number of significant SNPs contained in the genomic region was also calculated. If the number of SNPs in corresponding genomic region was more than 10, these regions were defined as peaks or genomic regions. The most significant SNP means the peak SNP.”*

Specific issues:

Introduction:

L70: is critical for

*Authors’ response 5: We modified the corresponding part accordingly. The modifications in the manuscript (line 89 in the revised manuscript with track changes).*

L84-87: This paragraph should be integrated into the previous paragraph?

*Authors’ response 6: We combined these two paragraphs according to the suggestion. The modifications in the manuscript (line 91-101 in the revised manuscript with track changes).*

L88-92: Please provide more introduction for the significant novel findings of this work.

*Authors’ response 7: We provided more information of the novel findings in the introduction part (line 107-121, line 684-742 in the revised manuscript with track changes).*

*The modifications in the manuscript (line 107-121 in the revised manuscript with track changes): “Although several studies identifying quantitative trait loci (QTLs) associated with spike morphology traits have been reported, few studies have focused on more than ten traits in a single study. Here, we conducted a large-scale GWAS based on approximately 40 million filtered SNPs for 27 spike morphology traits to dissect understand the genetic basis pf spike morphology. This approach may save a lot of time and work to get relatively precise locations of candidate genes leading to fast cloning of those genes for economically important traits. The genotype–phenotype map of these 27 traits revealed novel genomic regions associated with each putative trait and candidate genes. The 27 spike morphology traits enabled us to identify 392 additional genomic regions, indicating the value of dissecting spike morphology genetics for the detection of novel signals. Through GWAS, we identified and validated the gene SQUAMOSA PROMOTER BINDING PROTEIN-LIKE17 (TaSPL17), whose product positively regulates grain yield through grain number and size. Our results provide insights into the genetic basis of wheat spike morphology traits and serve as a resource for the genetic improvement of wheat grain yield.”*

*The modifications in the manuscript (line 675-729 in the revised manuscript with track changes): “This study achieves two significant objectives: first, we developed a large-scale phenotype-genotype map of 27 spike morphology traits for 306 worldwide wheat accessions; second, we clarified the mechanism of TaSPL17 regulating grain number and size in wheat. In addition, we elaborated on the geographical differentiation and breeding selection of TaSPL17 haplotypes and their value in wheat breeding. For the first objective, we carried out GWAS using 40 million SNPs. Although a substantial number of wheat GWAS has been previously conducted, many of these studies on complex traits were underpowered owing to the relatively small number of SNPs used (<1 million).*

*Several studies reported that LD for wheat genome was above 1Mb (r2=0.5) [24, 48, 49]. In this study, based on r2= 0.5, LD was 0.32Mb, 0.007Mb, 0.19Mb and 0.116Mb for A, B, D and whole genome, respectively. This observation is due to the strong genomic variation among the 306 worldwide wheat accessions. Traditionally, fine-mapping of a candidate gene in wheat would involve the development of several genetic populations and the identification of sufficient markers to obtain good genomic coverage, which may take several years. The large number of SNPs (~40 million SNPs, 2.8 SNPs per kb) and low LD used in this study allowed us to identify SNPs that are within or proximal to the candidate genes. This information may make it easier to obtain relatively precise locations of candidate genes.*

*Previous work identified very few major-effect genomic regions for grain yield, since grain yield is associated with grain number and size, which are influenced by different factors. We thus opted to dissect grain weight, number and size using 27 traits. The GWAS for assimilate partitioning traits revealed strong signals that would not have been observed with GWAS conducted directly on grain yield, number or size. The haplotypes at the identified peaks exhibited significant effects for the traits of grain number and size. The dissection of spike morphology using 27 traits allowed us to detect additional peaks, which is a powerful strategy to detect signals for grain yield traits in wheat.*

*Spikelet number per spike and grain number per spikelet are two determinants of grain number per spike in wheat. In addition, the “paired spikelet” is another promising trait for improving wheat spikelet number [50-53]. Spikelet number per spikelet is relatively stable in variable environments. Although a relatively large number of genes regulating spikelet number has been investigated [54-59], few studies have been reported in wheat on the molecular mechanism of floret fertility/grain number per spikelet [60], because grain number per spikelet exhibits high sensitivity in variable environments. In this study, we identified a gene regulating floret fertility/grain number per spikelet in wheat. The tradeoff between grain number and size negatively influences grain yield in wheat [61, 62]. We found that overexpressing TaSPL17 increased grain number per spikelet and TGW, overcoming the tradeoff between these two determinants of grain yield in wheat.”*

Results:

L139: Please provide the reference for the previous work in this sentence.

*Authors’ response 8: We provided a recent review as the reference according to the suggestion (line 169 in the revised manuscript with track changes).*

L146-148 and L152-171: There are too many introductions and explanations for these traits, which should be written into the sections of introduction or methods.

*Authors’ response 9: We removed some introductions and explanations of these traits to methods part (line 805-838 in the revised manuscript with track changes).*

L178: I cannot find the information of these significant marker-trait associations across the three subgenomes in Figure 2A-D.

*Authors’ response 10: We removed Figure 2A-D accordingly. In the revised manuscript, we added a new Figure 2, Figure S5 and Table S6 to show the genotype-phenotype map with the peaks for the corresponding traits in each chromosome.*

L199-200: It is more appropriate to discuss these contents in the discussion section.

*Authors’ response 11: We discussed these contents in the discussion section. The modifications in the manuscript (line 715-717 in the revised manuscript with track changes): “The dissection of spike morphology using 27 traits allowed us to detect additional peaks, which is a powerful strategy to detect signals for grain yield traits in wheat.”*

Line 203: It's not clear what are the "eight detailed traits", and what is the purpose describing them in this paragraph. Are these traits used for the identification of the candidate region in Chr7? More detailed explanations are needed.

*Authors’ response 12: According to the suggestions, we provided more explanations and the names of the eight detailed traits were shown in Figure S5 (line 309-333 in the revised manuscript with track changes, Additional file 2: Figure S5).*

*The modifications in the manuscript (line 309-333 in the revised manuscript with track changes): “To further validate the power of the assimilate partitioning traits for detecting significant associations in GWAS, we present GWAS data for eight traits related to assimilate partitioning between spike components. These eight traits magnify the existing variation among different wheat accessions, allowing us to observe strong signals associated with grain weight and number. We detected different peaks for the eight traits on different chromosomes (Additional file2: Figure S5), including peaks for awn weight. OsSPL14 in rice (Oryza sativa) (SQUAMOSA PROMOTER BINDING PROTEIN-LIKE14, also named as IDEAL PLANT ARCHITECTURE1 [IPA1]) is involved in the regulation of spike architecture, spikelet number and tillering [31-34]. OsSPL14 is a new "Green Revolution" gene that plays an important role in regulating plant architecture in rice [35-37]. HvSPL14 in barley (Hordeum vulgare) is associated with very similar spike phenotypes for its KO lines [38]. TaSPL17 is orthologous to OsSPL14 [39, 40], however, its effects on wheat plant architecture and grain yield remain largely unknown. One identified genomic region (225,328,984 bp to 225,737,586 bp) contained TaSPL17-A (TraesCS7A02G246500) and another two genes (TraesCS7A02G246400, TraesCS7A02G246300) (Fig 3a, Additional file2: Figure S5). We extracted the relative transcript levels of the three genes at six developmental stages according to inflorescence morphological features from a published transcriptome dataset of wheat spike [41, 42]. Only one gene (TraesCS7A02G246500) was expressed at high levels during spikelet and floret initiation and development, making it a high-confidence candidate, as the other two genes were almost not detectable during this phase (Fig. 3b).”*

L206-208: As mentioned above, please provide more explanation for why you selected this genomic region on chromosome 7A from 253 regions with strong GWAS signals?

*Authors’ response 13: According to the suggestion, we provided more explanation for the selection of this genomic region (line 309-333 in the revised manuscript with track changes).*

L295 and L310: Figure 3 include only A-L, and where is the Figure 3M?

*Authors’ response 14: Thank you! We modified the manuscript accordingly (line 422 in the revised manuscript with track changes).*

L294-295: Please describe the differences in plant architecture and spike morphology between WT and OE lines more detail.

*Authors’ response 15: We provided more details for the differences in plant architecture and spike morphology between WT and OE lines (line 425-439 in the revised manuscript with track changes).*

*The modifications in the manuscript (line 425-439 in the revised manuscript with track changes): “Plant height for the two OE lines was significantly shorter than that of WT (87.78 cm, WT; 79.42 cm, OE-A; 78.20 cm, OE-D; P <0.05); however, the lines showed no obvious differences in tiller number (8.20, WT; 7.10, OE-A; 7.40, OE-D) (Additional file 1: Table S11). The two OE lines had increased longer spikes and more grain per spike by 4.48-5.78% (0.52-0.67cm) and 11.21-13.21% (7.30-8.60), respectively (Fig. 4e, h, Additional file 1: Table S11). Although we observed no significant difference between spikelet number per spike and grain number per spikelet (central spikelet) between OE-A and WT, apical and basal spikelets did have more grains in OE-A, which may explain the increased grain number per spike. The higher values measured for grain width and length resulted in an increase in TGW of 8.81-11.27% (3.61-4.62 g) (Fig. 4i–k, Additional file 1: Table S11) in the two OE lines relative to WT. The higher grain number per spike and higher TGW together with the unchanged tiller number led to a 14.05-17.23% (1.84-2.26 g) increase in grain weight per plant in the two OE lines (Fig. 4m, Additional file 1: Table S11).”*

L298-299: Did you mean that plant height for the two OE lines was significantly shorter than that of WT?

*Authors’ response 16: Yes, we clarified it accordingly (line 425-427 in the revised manuscript with track changes).*

*The modifications in the manuscript (line 416-420 in the revised manuscript with track changes): “Plant height for the two OE lines was significantly shorter than that of WT (87.78 cm, WT; 79.42 cm, OE-A; 78.20 cm, OE-D; P <0.05);”*

L333-335: the terminal spikelet stage and anthesis stage were promoted in OE lines, but delayed in KO lines. We conclude that overexpression of HGY1 accelerate spikelet/floret initiation and development.

*Authors’ response 17: We modified the contents accordingly (line 464-470 in the revised manuscript with track changes).*

*The modifications in the manuscript (line 455-461 in the revised manuscript with track changes): “Compared to WT, the terminal spikelet stage (TS), yellow anther stage (YA), heading stage (HD), and anthesis stage (AN) were promoted in OE lines, but delayed in KO lines (Additional file 2: Figure S10). We concluded that overexpression of TaSPL17 accelerates spikelet/floret initiation and development.”*

L391: between -> among

*Authors’ response 18: We modified the contents accordingly. The modifications in the manuscript (line 533 in the revised manuscript with track changes).*

Lines 410-412: The changes of allelic frequencies are not sufficient for positive or negative selection of haplotypes. More careful population genetic analyses are needed to draw a valid conclusion. For example, Tajima's D or other tests.

*Authors’ response 19: Thanks for the kind suggestion. We conducted further analyses to support the conclusion (line 557-559 in the revised manuscript with track changes).*

*The modifications in the manuscript (line 547-548 in the revised manuscript with track changes): “A Tajima’s D value of 4.16 for the 799 Chinese wheat accessions using VCFtools indicated that these groups were subject to balanced selection [43].”*

Figure 6: It would be more convincing to provide photographs to show the phenotypes of the RILs and NILs.

*Authors’ response 20: We provided photos for spikes of NILs and RILs (Additional file 2: Figure S11).*

*Figure S11. The spikes for NILs and RILs of TaSPL17 in field. TaSPL17-A-L and TaSPL17-A-H indicate the lines with low (L) and high (H) alleles for the corresponding traits. The first and second rows show the front and side of the spikes for TaSPL17-A-L and TaSPL17-A-H.*

L472: Please describe the difference between these two combinations more detailed.

*Authors’ response 21: Thanks for the kind suggestion. We provided more details for the two combinations (line 619-624 in the revised manuscript with track changes).*

*The modifications in the manuscript (line 619-624 in the revised manuscript with track changes): “We observed the most marked differences between the two combinations Hap-A3+Hap-B3 and Hap-A2+Hap-B1 (Additional file 1: Table S12). Notably, compared to Hap-A3+Hap-B3, the combination Hap-A2+Hap-B1 had increased grain number per spike, grain number per spikelet, and spikelet density by 13.35% (7.10), 7.83% (1.84) and 21.44% (0.51), respectively (Additional file 1: Table S12).”*

Figure 5G: The axis labels are missing.

*Authors’ response 22: We added the axis labels accordingly.*

Discussion:

L529: The structure of discussion should be further improved to highlight the most significant contribution and novel findings of this work.

*Authors’ response 23: Thanks for the kind suggestion. We modified the discussion according to the suggestion.*

*The modifications in the manuscript (line 684-786 in the revised manuscript with track changes).*

L555-568: As mentioned above, the authors just present the previous work in this paragraph. It would be nice to discuss and compared the roles of these genes in grain yield.

*Authors’ response 24: Thanks for the kind suggestion. We modified the discussion according to the suggestion (line 705-729 in the revised manuscript with track changes).*

*The modifications in the manuscript (line 705-729 in the revised manuscript with track changes): “Spikelet number per spike and grain number per spikelet are two determinants of grain number per spike in wheat. Spikelet number per spikelet is relatively stable in variable environments. A relatively large number of genes regulating spikelet number has been investigated [48-53]. In addition, the “paired spikelet” is another promising trait for improving wheat spikelet number [54-57]. However, few studies for the molecular mechanism of floret fertility/grain number per spikelet have been reported in wheat [58], because grain number per spikelet exhibits high sensitivity in variable environment. In this study, we cloned a gene regulating floret fertility/grain number per spikelet in wheat. The tradeoff between grain number and size negatively influences grain yield in wheat [59, 60]. We found that overexpressing TaSPL17 increased grain number per spikelet and TGW, overcoming the tradeoff between these two determinants of grain yield in wheat.”*

Supplementary Table S1: Latin names of species should be italicized.

*Authors’ response 25: Thanks for the kind suggestion. We changed the names accordingly.*

Reviewer #2:

This manuscript reported the GWAS analysis for traits related to assimilate distribution between grains and spike components. A candidate gene High Grain Yield1 (HGY1), encoding a SPL transcription factor was identified to positively control grain size and number by regulating the development of spikelet and floret meristems, resulting in enhanced grain weight per plant. The work about HGY1 function and utilization in wheat breeding is well planned and informative. However, the GWAS part does not make sense. My major concerns are:

1. The authors claimed that GWAS of assimilate partitioning between grains and spike chaff is a powerful strategy to detect signals for grain yield traits in wheat. I disagree with this conclusion due to a couple of concerns.

1) Lines 145-146: We observed strong signals in the GWAS results for these traits that cannot be revealed by GWAS conducted directly on grain yield, number or size. It is not reasonable and understandable that none of genomic locus was associated with grain yield, number or size. Also, it is meaningless for genomic regions related to dissected traits (13 traits in this MS) but not related to grain yield, number or size.

*Authors’ response 1: Thanks for the kind suggestion. We included 14 spike morphology/grain yield traits (27 traits in total) for the 306 wheat accessions to verify the conclusion that GWAS of assimilate partitioning between grains and spike chaff is a powerful strategy to detect signals for grain yield traits in wheat. The GWAS results of the 27 spike morphology traits make the conclusion more clearly. There are differences and overlaps of genomic loci between assimilate partitioning traits and grain yield traits. The genomic loci that were only identified by assimilate partitioning traits also influence grain yield, number, or size (line 264-289 in the revised manuscript with track changes).*

*The modifications in the manuscript (line 265-289 in the revised manuscript with track changes): “With the set of 40,710,923 SNPs defined above (MAF > 0.05; missing rate < 20%; missing genotype rate < 0.1), we performed GWAS for 27 spike morphology traits. We identified 132,086 significant marker–trait associations (–Log10[P value] > 5.0) across the three subgenomes, with 33,619 associations from the A genome, 90,158 from the B genome, and 8,309 for the D genome (Additional file 1: Table S5). We also used LD and connections between markers to delineate 590 genomic regions associated with the 27 traits, comprised of 216 for the A genome, 254 for the B genome, and 120 for the D genome, (Fig 2, Additional file 1: Table S6). Of the 590 identified genomic regions, 422 were associated with more than one trait. Some associated genomic regions exhibited pleiotropic effects on the traits related to grain number/size and assimilate partitioning, while the genomic regions for grain number/size traits were not as strong as the regions for assimilate partitioning traits. For instance, we identified one region (1B: 628,535,896-629,920,890) when mapping for grain weight/rachis weight (entire spike) and spikelet density. The best SNP for the signal of grain weight/rachis weight (entire spike) (assimilate partitioning) were of much higher significance (–Log10[P value] =9.37) than the best SNP of spikelet density (–Log10[P value] =6.07). In addition, 392 peaks associated with assimilates distribution (ratios between spike component weight) were not observed for the remaining spike morphology/grain yield traits (Additional file 1: Table S6, S7). Haplotype analyses showed that 296 of 392 peaks have effects on the remaining spike morphology/grain yield traits (Additional file 1: Table S7). These results suggest that the ratios between the weights of spike components minimize the variance among the values of spike components, which in turn maximize the ability to detect differences for that spike component. This approach increases the power of GWAS to detect significant associations and highlights the necessity of conducting GWAS on assimilate partitioning traits.”*

2) All of 13 traits are ratios of two agronomy traits, some of which indicated the assimilate distribution between grains and spike. Although these ratios were associated with grain yield, the ratio was not a good choice for GWAS analysis. Does the ratio normally distribute? KO lines showed decreased grain number and grain weight as shown in Figure 2, but they showed higher grain number/spike chaff and grain weight/chaff as shown in Supplemental Figure S4. Thus, ratio of grain number to spike chaff etc was not meaningful.

*Authors’ response 2: Thanks for the kind suggestion. The assimilate partitioning traits provided a new choice for GWAS analysis. Our work revealed that the GWAS results of assimilate partitioning traits can identify some additional signals, which are different from the GWAS of grain yield, number or size. The ratios normally distribute (Additional file 2: Figure S3). KO lines negatively affect both grains and chaff. Our conclusion is that the higher grain number/spike chaff and grain weight/chaff maybe due to the different sensitivities of grains and spike chaff to the knockout of this gene. This is consistent with the finding that some signals affecting grain number/size and assimilate partitioning traits were detected by GWAS of assimilate partitioning traits, but these signals were not observed in the GWAS results of grain number and size traits. We added more explanations for this part (line 402-409 in the revised manuscript with track changes). The ratio of grain number to spike chaff is defined as fruiting efficiency in previous studies (line 791-793 in the revised manuscript with track changes).*

*The modifications in the manuscript (line 402-409 in the revised manuscript with track changes): “Notably, KO lines had decreased grain number and grain weight (Fig. 2j, m), but they showed higher grain number/spike chaff and grain weight/chaff (Additional file 2: Figure S8). KO lines negatively affected both grains and chaff. We speculate is that the higher grain number/spike chaff and grain weight/chaff maybe due to different sensitivities of grains and spike chaff to the knockout of this gene. This is consistent with the finding that some signals affecting grain number/size and assimilate partitioning traits were detected by GWAS of assimilate partitioning traits, but these signals were not observed in the GWAS results for grain number and size traits.”*

*The modifications in the manuscript (line 805-807 in the revised manuscript with track changes): “Fruiting efficiency is often calculated at physiological maturity as the grain number per unit of chaff dry weight and has been proposed as a target trait to improve grain number and yield potential [75-77].”*

*Fig. 2 The genotype–phenotype map for the 27 spike morphology traits. The genotype–phenotype map with the peaks for the corresponding traits in each chromosome aligned to the reference sequence of bread wheat. Entire spike means that the traits were determined based on the entire spike. Individual spikelet was collected from the center of spike. The symbol “/” indicates the ratio between the two traits.*

*Figure S3. The distribution of phenotypic values of the 27 spike morphology traits for the 306 wheat accessions. The blue, red and green colors indicates the traits of the entire spike, the traits of individual spikelet, and the traits of ratios between spike components. The symbol “/” indicates the ratio between the two traits.*

3) The genomic region (chromosome 7A: 225.33 Mb) was associated with 8 traits including Awn weight per spike, Awn weight/grain weight, Awn weight/spike chaff weight, Chaff weight per spikelet/spikelet weight, Grain number/spike chaff weight (entire spike), Grain weight per spikelet/spikelet weight, Grain weight per spikelet/spikelet weight? How to explain that the SPL TF affected awn weight?

*Authors’ response 3: Thanks for the kind suggestion. The SPL influence spike morphology traits, including grains and spike chaff. KO lines negatively affect both grains and chaff. The awn weight is an important part of spike chaff weight (line 213-215 in the revised manuscript with track changes).*

*The modifications in the manuscript (line 213-215 in the revised manuscript with track changes): “Awn weight per spike was strongly and positively associated with spike chaff weight, suggesting that awn is an important part of spike chaff.”*

4) TaSBEIIa, involved in starch biosynthesis, should be related to grain weight, but it was associated with grain number per spikelet/chaff weight per spikelet in this study (Supplemental Table S7). WAPO1 should be related to spikelet number per spike, but it is associated with grain weight/rachis weight (entire spike) in this study (Supplemental Table S7). The GWAS analysis in this MS is not supportive.

*Authors’ response 4: Thanks for the kind suggestion. Based on these results, we would like to provide some new information, which indicate that these genes may influence other traits. Due to the comments, to avoid any misunderstanding, we removed these two genes for these two traits.*

2. These 306 wheat accessions were mainly landraces collected from more than 70 countries. I wonder why the modern wheat cultivars showing elite agronomy traits were not included in this population.

*Authors’ response 5: Thanks for the kind suggestion. There are two reasons for the selection of the 306 accessions. 1. The spike morphology traits displayed great differences among landraces collected from more than 70 countries (Figure S2, S3), which enabled us to detect strong signals. 2. we would like to detect some genes, which were not widely used in modern wheat cultivars. Indeed, we found that the desired alleles of the SNP in the candidate gene have been lost in the past century (Fig. 6g).*

3. Phenotypic Analyses of this population including phenotypic variation range, heritability, mean for all traits should be added in the results part and supplemental data. If the traits (ratios) were not normally distributed, GWAS was not available.

*Authors’ response 6: According to the suggestions, we included the phenotypic range, heritability, mean for all the traits in the results part and supplemental data (Additional file 1: Table S4). All the traits were normally distributed with three exceptions: awn weight per spike, awn weight/grain weight, awn weight/spike chaff weight (Figure S3). The abnormal distribution of awn weight may attribute to the geographical differentiation, which enabled us to identify a very strong signal (1057 marker-trait associations), including Awn Length Inhibitor 1 (ALI-1, TraesCS5A02G542800).*

*The modifications in the manuscript (line 187-195 in the revised manuscript with track changes): “All traits followed a normal distribution with three exceptions: awn weight per spike, awn weight/grain weight, and awn weight/spike chaff weight (Additional file 2: Figure S3). The abnormal distribution of these traits may be attributed to the geographical differentiation of wheat awn. Interestingly, these three traits had relatively high heritability among the 27 traits (awn weight/spike weight, h2=0.87; awn weight/grain weight, h2=0.73; awn weight/spike chaff weight, h2=0.72) (Additional file 1: Table S4). The broad genotypic variation and relatively high heritability of most traits (Additional file 1: Table S4) suggests the large potential and a genetic basis for the increase in grain yield by manipulating spike morphology.”*

4. A total of 113,413 significant marker-trait associations were identified. Among such a huge scale of candidate loci, how to select this genomic region (chromosome 7A: 225.33 Mb) including only 20 marker-trait associations for further analysis?

*Authors’ response 7: According to the suggestion, we provided more details for the selection of this genomic region (line 309-333 in the revised manuscript with track changes).*

*The modifications in the manuscript (line 309-333 in the revised manuscript with track changes): “To further validate the power of the assimilate partitioning traits for detecting significant associations in GWAS, we present GWAS data for eight traits related to assimilate partitioning between spike components. These eight traits magnify the existing variation among different wheat accessions, allowing us to observe strong signals associated with grain weight and number. We detected different peaks for the eight traits on different chromosomes (Additional file2: Figure S5), including peaks for awn weight. OsSPL14 in rice (Oryza sativa) (SQUAMOSA PROMOTER BINDING PROTEIN-LIKE14, also named as IDEAL PLANT ARCHITECTURE1 [IPA1]) is involved in the regulation of spike architecture, spikelet number and tillering [31-34]. OsSPL14 is a new "Green Revolution" gene that plays an important role in regulating plant architecture in rice [35-37]. HvSPL14 in barley (Hordeum vulgare) is associated with very similar spike phenotypes for its KO lines [38]. TaSPL17 is orthologous to OsSPL14 [39, 40], however, its effects on wheat plant architecture and grain yield remain largely unknown. One identified genomic region (225,328,984 bp to 225,737,586 bp) contained TaSPL17-A (TraesCS7A02G246500) and another two genes (TraesCS7A02G246400, TraesCS7A02G246300) (Fig 3a, Additional file2: Figure S5). We extracted the relative transcript levels of the three genes at six developmental stages according to inflorescence morphological features from a published transcriptome dataset of wheat spike [41, 42]. Only one gene (TraesCS7A02G246500) was expressed at high levels during spikelet and floret initiation and development, making it a high-confidence candidate, as the other two genes were almost not detectable during this phase (Fig. 3b).”*

5. How were the KO lines with single and double knocking out obtained, as three homoeologous genes were recognized by single guide RNA?

*Authors’ response 8: Thanks for the kind suggestion. For CRISPR/Cas9 genome editing in wheat, one or two guide RNAs were usually designed in the conserved region of three homoeologous genes, it has a chance to isolate different types of triple mutants, double mutants and single mutants in transgenic progenies. We added the information of different generations of KO lines in Table S9.*

6. the results about HGY (SPL) affecting spike morphology and grain size were interesting and the evidence were solid. But the connection between GWAS and HGY functional analysis were weak.

*Authors’ response 9: According to the suggestions, we provided more details to show the connection between GWAS and the candidate gene functional analysis (line 264-289, 309-333 in the revised manuscript with track changes, in Authors’ response1, 9).*

Minor Concerns:

1) Wrong spelling for "homeologous". It should be homoeologous.

*Authors’ response 10: Thanks. We changed it accordingly.*

2) Line 38: encoding a transcription factor of the SQUAMOSA promoter-binding protein-like family. Family should be deleted.

*Authors’ response 11: Thanks. We deleted “family”.*

3) Supplemental Figure S3: HGY1 was shown as TaSPL17, which will make the reader confused.

*Authors’ response 12: Thanks. We changed HGY1 to TaSPL17 across the manuscript.*

4) This MS was bad-written and should be improved by native English speaker.

*Authors’ response 13: Thanks for the kind suggestion. We sent the manuscript to language editing service. Please find the certificate below. We also included it in the attachment.*

Reviewer #3:

This manuscript by Liu et al. provides an interesting advance in our understanding of the genetic control of yield related traits in wheat, and introduces us to the potential importance that a new gene in the form of an SPL transcription factor could play in improving the yield of wheat. In particular, the phenotyping approach used by the authors provide a new method to break down the complexity of yield as a trait, and the GWAS approach provides an excellent example of how new genomic sequence information could be used to uncover beneficial genetic variation in breeding programs. The paper included an excellent spread of molecular and genetic characterisation of this SPL transcription factor, and the potential yield improvements are considerable. The manuscript should have broad interest across plant sciences, breeding and biotechnology. I do have a few concerns which are outlined below.

MAJOR CONCERNS

L252-255: Can the authors please include the information in the main text about the gene edits that occurred in the transgenic lines, for each of the homeologs, and in doing so, provide more information about the nature of the "knock-outs"? For example, are they small deletions or insertions (if so, what size), are they missense mutations, deletions that cause frameshift or nonsense mutations? If they are small deletions and insertions, what is the consequence of these changes?

*Authors’ response 1: Thanks. We have added the information according to the suggestions (line 366-369 in the revised manuscript with track changes).*

*The modifications in the manuscript (line 375-378 in the revised manuscript with track changes): “A 1-bp deletion and a 1-bp insertion were most frequently identified at the target site. All mutations caused a shift in the reading frame and introduced premature translation termination codons (Additional file 1: Table S9, Additional file 2: Figure S6).”*

L277-278: To me, the gene nomenclature appears to be a contraction based on the name being derived from the KO lines, which clearly show reduced grain yield. Therefore, the gene name appears to refer to the wild-type, which is not high grain yield, but just relatively high compared to the knock-outs. I recommend the authors either better explain the basis of the gene nomenclature, or they change the gene name to reflect the phenotype of the knock-out lines, which follows convention.

*Authors’ response 2: According to the suggestion, we changed the name to TaSPL17. Meanwhile, we provided the explanations for the gene nomenclature (line 304-320 in the revised manuscript with track changes).*

*The modifications in the manuscript (line 313-339 in the revised manuscript with track changes): “We detected different peaks for the eight traits on different chromosomes (Additional file2: Figure S5), including peaks for awn weight. OsSPL14 in rice (Oryza sativa) (SQUAMOSA PROMOTER BINDING PROTEIN-LIKE14, also named as IDEAL PLANT ARCHITECTURE1 [IPA1]) is involved in the regulation of spike architecture, spikelet number and tillering [31-34]. OsSPL14 is a new Green Revolution gene that plays an important role in regulating plant architecture in rice [35-37]. HvSPL14 in barley (Hordeum vulgare) is associated with very similar spike phenotypes for its KO lines [38]. TaSPL17 is orthologous to OsSPL14 [39, 40], however, its effects on wheat plant architecture and grain yield remain largely unknown. One identified genomic region (225,328,984 bp to 225,737,586 bp) contained TaSPL17-A (TraesCS7A02G246500) and another two genes (TraesCS7A02G246400, TraesCS7A02G246300) (Fig 3a, Additional file2: Figure S5). We extracted the relative transcript levels of the three genes at six developmental stages according to inflorescence morphological features from a published transcriptome dataset of wheat spike [41, 42]. Only one gene (TraesCS7A02G246500) was expressed at high levels during spikelet and floret initiation and development, making it a high-confidence candidate, as the other two genes were almost not detectable during this phase (Fig. 3b). The distance between TraesCS7A02G246500 and the lead SNP was less than 400 kb (Additional file 1: Table S8). The homoeologous genes of TraesCS7A02G246500 (Fig. 3c) are TraesCS7B02G144900 (TaSPL17-B, B genome) and TraesCS7D02G245200 (TaSPL17-B, D genome).”*

L333-334: I don't agree with some of the conclusions made in this section, particularly that the KO lines have a delayed phase transition. For example, the KO line arrives at the TS stage at the same time as the WT. I agree that it flowers later, and therefore the time between TS and anthesis may be different, but without analysis of developmental stages in between TS and AN, I would be cautious about saying there is a phase transition phenotype. Also, did the OE-A line show a similar phenotype to OE-D for the data shown in Supp Figure 6?

*Authors’ response 3: Thanks. According to the suggestion, we included the data of another two stages in between TS and AN stage (line 454-461 in the revised manuscript with track changes). Instead of saying there is a phase transition phenotype, we clarified that “the KO/OE lines also showed clear effects on spike development process”. OE-A line showed similar phenotypes to OE-D, we added these data accordingly (Figure S10).*

*The modifications in the manuscript (line 464-466 in the revised manuscript with track changes): “Compared to WT, the terminal spikelet stage (TS), yellow anther stage (YA), heading stage (HD), and anthesis stage (AN) were promoted in OE lines, but delayed in KO lines (Additional file 2: Figure S10). We concluded that overexpression of TaSPL17 accelerates spikelet/floret initiation and development.”*

L348: Regarding the in-situ hybridisation experiments, I would like to know if the authors can confidently say that the probe only bound to HGY-D1? The reason for asking this is that given probe sequences can be very similar between homeologs, I suspect that a HYG-D1 probe will also bind to HYG-A1 and HYG-B1. It may therefore be more accurate to state HYG-1 as the probe, rather than HYG-D1.

*Authors’ response 4: Thanks for the kind suggestion. Thanks for the suggestion. We have revised the description of the probe to HYG-1/TaSPL17 according to the suggestion (line 481-487, 983 in the revised manuscript with track changes).*

I have a few concerns regarding the haplotype analysis, especially regarding the assignment of causality. For example, 1) L385-388: based on what data are the authors concluding that Hap-A2 increases spikelet number, decreased spike length and increased grain number without having generated near-isogenic lines. These conclusions seem too strong based on the presented evidence. The same comment goes for L445-446 for HYG-B1. 2) In describing the haplotypes, it would help future studies if the authors made it clear which haplotype is most like the reference genome sequence. 3) In describing the haplotypes, it would help to understand if the phenotypes of these alleles, and the consequence of the sequence changes, fits with the data coming from the transgenic lines. For example, we would expect Hap-A2 has a gain-of-function affect to fit with the OE lines. Is this the case?

*Authors’ response 5: Thanks for the kind suggestion. (1) We modified the conclusion according to the suggestions (line 526-530, 584-587 in the revised manuscript with track changes). (2) Hap-A1 had the same sequence to reference genome (Chinese Spring) (line 539-540). Hap-B1 and Hap-B2 exhibited high similarity to reference genome relative to Hap-B3 and Hap-B4 (line 592-593). We clarified the similarity of haplotypes to the reference genome sequence in the manuscript. (3) Although the positive effect of Hap-A2 and OE lines are identical, however the effects of OE lines are much stronger relative to Hap-A2.*

*The modifications in the manuscript (line 526-530 in the revised manuscript with track changes): “Compared to Hap-A1, the wheat accessions harboring Hap-A2 had higher average value of spikelet number per spike by 8.85% (1.88) but lower average value of spike length by 7.30%, (0.80 cm), leading to a higher spikelet density of 19.08%. Hap-A2 also had higher average value of grain number by 13.78%, (6.25) (Fig. 6b–e, Additional file 1: Table S12).”*

*The modifications in the manuscript (line 584-587 in the revised manuscript with track changes): “Of these four haplotypes, the wheat accessions harboring Hap-B4 had the highest average value of spikelet number per spike, and grain number per spikelet, resulting in the highest grain number per spike of all Hap-B haplotypes (Additional file 1: Table S12).”*

Following on from this, it is not clear which allele is used for the RILs and NILs from L488-527. For example, when the authors stage "we introgressed the HYG1 allele conferring high spikelet number...", what allele are they referring to and what haplotype/homeolog does this correspond to? In reference to the Bainong64 vs Jingshuang16 allele, what is the predicted functional consequence of this missense mutation? Is it in a domain of importance? Is it predicted to be deleterious? More information would help here.

*Authors’ response 6: Thanks for the kind suggestion. The nonsynonymous SNP locates at +4,245 bp in the gene, +1,046 bp in the cDNA. The nonsynonymous SNP altered one amino acid (+316 protein sequence, Glu/Asp) from aspartate (Bainong64) to glutamate (Jingshuang16). The nonsynonymous SNP is not in the conserved domain, it is not the target of microRNA. Based on the current results, it is difficult to conclude the mechanism of the effects of this SNP on the corresponding traits.*

*The modifications in the manuscript (line 640-643 in the revised manuscript with track changes): “To relate the contributions of TaSPL17 alleles to grain yield traits and evaluate the value of TaSPL17 in wheat breeding, we introgressed the TaSPL17 allele (A/G at +4,245 bp in the gene, +1,046 bp in the cDNA) conferring high spikelet number, grain number per spikelet and grain size to improve the potential of grain yield.”*

*The modifications in the manuscript (line 638-643 in the revised manuscript with track changes): “We sequenced the coding sequence of TaSPL17-A in multiple accessions and detected a nonsynonymous SNP (A/G at +4,245 bp in the gene, +1,046 bp in the cDNA) between Bainong64 (an elite wheat cultivar widely planted in China) and Jingshuang16. The nonsynonymous SNP altered one amino acid (+316 protein sequence, Glu/Asp) from aspartate (Bainong64) to glutamate (Jingshuang16)”.*

The authors should include a section in the materials and methods explaining the statistical tests used in the paper.

*Authors’ response 7: Thanks. According to the suggestion, we included a section in the materials and methods explaining the statistical test.*

*The modifications in the manuscript (line 917-938 in the revised manuscript with track changes):*

*Multiple comparisons and Best linear unbiased estimates*

*The analysis was carried out using the agricolae package in R (agricolae: Statistical Procedures for Agricultural Research. R package version 1.3-1.https://CRAN.R-project.org/package=agricolae). The means of multiple groups were compared using the best significant difference (LSD) test, which required a one-way ANOVA.*

*The best linear unbiased estimates (BLUEs) for 27 traits across two years were calculated using the lme4 package in R [86] with the linear mixed linear model:*

*Yij =u +genotyi +Yearj + (genoty *Year)ij + errorij*

*Where Yij is the trait of the i-th genotype in j-th year; u is the mean; genotyi is the genotype effect of the i-th genotype; Yearj, is the effect of the j-th year; (genoty *Year)ij is the genotype-year interaction; errorij is the error of the j-th year and items were set to random.*

*Broad sense heritability*

*Phenotypic data from all environments were analyzed by analysis of variance (ANOVA) in R. Broad sense heritability (H2) of 27 traits was calculated across environments from variance components according to the formula:*

*H2= (σG2)/(σG2+(σGE2)/2+(σe2)/2r)*

*Where σ_G^2 is the genotypic variance, σ_GE^2 is the genotype by the year effect, σ_e^2 is the residual error, and r is the average number of replications (r=5).*

MINOR CONCERNS

Line 219: correct to "TraesCS7A02G24600 are TraesCS7B02G144900" instead of "TraesCS7A02G24600 were TraesCS7B02G144900".

*Authors’ response 8: We changed it accordingly (line 337 in the revised manuscript with track changes).*

L298: Do the authors mean Fig. 3D here rather than 3E?

*Authors’ response 9: It is Fig. 3D. We changed it accordingly (line 424 in the revised manuscript with track changes).*

Figure 3: Are all of these images and data from plants grown in the glasshouse? Please include this detail in the figure legend if so.

*Authors’ response 10: Thanks. They are from plants grown in field. We included this information (353, 354, 444 in the revised manuscript with track changes).*

L329: Why do authors refer to Supp Figure S4? Do they mean S5, here?

*Authors’ response 11: It is Figure S5. We changed it accordingly.*

For all of the column graphs used in this article, I suggest the authors include the individual data point that contribute to the average and SEM.

*Authors’ response 12: Thanks for the kind suggestion. We included the individual data point in Fig. 6b-e, we have more than 30 individual values for each column. For the remained figures, we only have 3-5 replicates for each trait, which means there are only 3-5 points for each column, it is wired to include 3-5 points for each column.*

In general, I found some of the results sections ended abruptly without summarising the outcome of a given set of experiments. For example, it would improve the readability to include summary sentences after L310, 357, etc.

*Authors’ response 13: Thanks. We included summary sentences according to the suggestions (line 439-441, 494-495 in the revised manuscript with track changes).*

L390: Please include the positional information for the amino acid change, e.g., PXXXL.

*Authors’ response 14: We included the positional information for the amino acid change (line 532-533 in the revised manuscript with track changes).*

L559: When talking about FT2, the authors should cite Gauley and Boden, 2021, as they used hexaploid wheat whereas Shaw et al used tetraploid wheat.

*Authors’ response 15: We cited the paper (Gauley and Boden, 2021) accordingly (line 722 in the revised manuscript with track changes).*

**Second round of review**

**Reviewer 1**

The authors have addressed all my concerns.

**Reviewer 3**

The authors have undertaken a considerable amount of work to revise the manuscript, and have taken onboard all of the reviewers' comments.

I have two remaining concerns for the authors to address.

1) L64-65: The word "obvious" is too definitive here, I strongly recommend replacing with the word "indicates" or "suggests".

2) The gene abbreviations are incorrect as per the soon to be published guidelines for wheat gene nomenclature, and existing conventional practices. The gene names should be written as SPL-17, with the homeologues named as SPL-17A, SPL-17B and SPL-17D.

3) L463-464: this new sentence does not make sense, as it contains poor grammar ("spike development process" is not correct). Please fix.

**Reviewer 4**

As requested by the editor, my review focus on the association studies in this manuscript. The manuscript describes a genome-wide association study with 40 million SNPs for 27 traits measured on 306 wheat accessions. Among the 40 million SNPs, 2,756,291 SNPs were considered for multiple test correction. The authors thought about the Bonferroni threshold of 0.05/2,756,291=1.81E-7 (-Log10(P)=7.74). However, the threshold was decreased to 5.0 (1E-5) for the purpose of reducing false negatives, and reference 90 (Li Y et al.) was cited for doing so. In fact, the referenced paper used 0.05/number of SNPs with Log10(P) above 7.0. It is unclear where is 5.0 come from. I agree with reviewer #2: "The GWAS part does not make sense." I also feel that the revised manuscript does not address the concern.

Furthermore, Bonferroni multiple test correction does not mean the division of 0.05 by the number of SNPs. It should include all tests, e.g., the number of SNPs times the number of traits in this study. The authors did not find signals on original traits of grain yield and number/size, and they derived more traits on their components. The more traits, the more associations, even by random chances. It is for sure that Bonferroni multiple test correction could be too conservative due to the linkage between SNPs and correlation among traits. However, a reasonable threshold should be provided, such as permutation.

If the authors have a concern about the statistical power of the study, enhancing the analytical method can make a dramatic difference. EMMAx has the same statistical power as the regular mixed linear model developed in 2005. They are both single locus models. Since then, several multiple loci models have been developed with improved statistical power, including MLMM, FarmCPU, and BLINK. The authors should focus on the improvement of statistical power instead of lowering the experiment-wise threshold without support.

In addition to the inappropriate experiment-wise threshold, the GWAS conducted in the study also invited false positives. Figure 1C suggests that G3 and G8 are isolated from the rest groups. The GWAS section lacks details if population structure or PCs (how many) were fitted as covariates. Even if they were, it is unclear how G3 and G8 were modeled. Fitting population structures or PCs as covariates does not fully model the isolation. The dummy variables (indicators) of these two groups can remove many spurious associations with SNPs that are specific to the two groups.

Some of the traits, especially the derived traits from division, exhibit outliers which are sources of false positives. The examples include Awn wight/spile chaff weight. The outliers should be removed or pulled back to an acceptable boundary.

**Authors Response**

**Point-by-point responses to the reviewers’ comments:**

**Reviewer 3**

The authors have undertaken a considerable amount of work to revise the manuscript, and have taken onboard all of the reviewers' comments.

I have two remaining concerns for the authors to address.

1) L64-65: The word "obvious" is too definitive here, I strongly recommend replacing with the word "indicates" or "suggests".

2) The gene abbreviations are incorrect as per the soon to be published guidelines for wheat gene nomenclature, and existing conventional practices. The gene names should be written as SPL-17, with the homeologues named as SPL-17A, SPL-17B and SPL-17D.

3) L463-464: this new sentence does not make sense, as it contains poor grammar ("spike development process" is not correct). Please fix.

**Reviewer 4**

As requested by the editor, my review focus on the association studies in this manuscript. The manuscript describes a genome-wide association study with 40 million SNPs for 27 traits measured on 306 wheat accessions. Among the 40 million SNPs, 2,756,291 SNPs were considered for multiple test correction. The authors thought about the Bonferroni threshold of 0.05/2,756,291=1.81E-7 (-Log10(P)=7.74). However, the threshold was decreased to 5.0 (1E-5) for the purpose of reducing false negatives, and reference 90 (Li Y et al.) was cited for doing so. In fact, the referenced paper used 0.05/number of SNPs with Log10(P) above 7.0. It is unclear where is 5.0 come from. I agree with reviewer #2: "The GWAS part does not make sense." I also feel that the revised manuscript does not address the concern.

Furthermore, Bonferroni multiple test correction does not mean the division of 0.05 by the number of SNPs. It should include all tests, e.g., the number of SNPs times the number of traits in this study. The authors did not find signals on original traits of grain yield and number/size, and they derived more traits on their components. The more traits, the more associations, even by random chances. It is for sure that Bonferroni multiple test correction could be too conservative due to the linkage between SNPs and correlation among traits. However, a reasonable threshold should be provided, such as permutation.

If the authors have a concern about the statistical power of the study, enhancing the analytical method can make a dramatic difference. EMMAx has the same statistical power as the regular mixed linear model developed in 2005. They are both single locus models. Since then, several multiple loci models have been developed with improved statistical power, including MLMM, FarmCPU, and BLINK. The authors should focus on the improvement of statistical power instead of lowering the experiment-wise threshold without support.

In addition to the inappropriate experiment-wise threshold, the GWAS conducted in the study also invited false positives. Figure 1C suggests that G3 and G8 are isolated from the rest groups. The GWAS section lacks details if population structure or PCs (how many) were fitted as covariates. Even if they were, it is unclear how G3 and G8 were modeled. Fitting population structures or PCs as covariates does not fully model the isolation. The dummy variables (indicators) of these two groups can remove many spurious associations with SNPs that are specific to the two groups.

Some of the traits, especially the derived traits from division, exhibit outliers which are sources of false positives. The examples include Awn wight/spile chaff weight. The outliers should be removed or pulled back to an acceptable boundary.

**Reviewer #3:**

The authors have undertaken a considerable amount of work to revise the manuscript, and have taken onboard all of the reviewers' comments.

I have two remaining concerns for the authors to address.

1）L64-65: The word "obvious" is too definitive here, I strongly recommend replacing with the word "indicates" or "suggests".

***Authors’ response 1****: We modified it according to the suggestion (line 47 in the revised manuscript with track changes).*

2) The gene abbreviations are incorrect as per the soon to be published guidelines for wheat gene nomenclature, and existing conventional practices. The gene names should be written as SPL-17, with the homeologues named as SPL-17A, SPL-17B and SPL-17D.

***Authors’ response 2****: The name was written as TaSPL17 according to previous publications (reference 1, 2, 3, 4).*

***References:***

1. *TaSPL13 regulates inflorescence architecture and development in transgenic wheat (Triticum aestivum L.)*
2. *Pleiotropic function of the SQUAMOSA PROMOTER-BINDING PROTEIN-LIKE gene TaSPL14 in wheat plant architecture*
3. *Wheat TaSPL8 Modulates Leaf Angle Through Auxin and Brassinosteroid Signaling*
4. *Molecular characterization and expression analysis of Triticum aestivum squamosa-promoter binding protein-box genes involved in ear development*

3) L463-464: this new sentence does not make sense, as it contains poor grammar ("spike development process" is not correct). Please fix.

***Authors’ response 3****: Thank you! We changed it to “the process of spike development” according to the suggestion (line 388-389 in the revised manuscript with track changes).*

**Reviewer #4:**

As requested by the editor, my review focus on the association studies in this manuscript. The manuscript describes a genome-wide association study with 40 million SNPs for 27 traits measured on 306 wheat accessions. Among the 40 million SNPs, 2,756,291 SNPs were considered for multiple test correction. The authors thought about the Bonferroni threshold of 0.05/2,756,291=1.81E-7 (-Log10(P)=7.74). However, the threshold was decreased to 5.0 (1E-5) for the purpose of reducing false negatives, and reference 90 (Li Y et al.) was cited for doing so. In fact, the referenced paper used 0.05/number of SNPs with Log10(P) above 7.0. It is unclear where is 5.0 come from. I agree with reviewer #2: "The GWAS part does not make sense." I also feel that the revised manuscript does not address the concern.

***Authors’ response 4****: Thank you for the suggestions and comments. We modified the manuscript accordingly (line 253-265 in the revised manuscript with track changes).*

***To be honest, we are always happy to answer the new suggestions, comments and concerns. However, we are not sure if we understand them in the right way. If we did not understand them in the right way, we are delighted to answer the futher questions and comments from the reviewer.***

*GWAS controls the probability of false positives, and the threshold is generally adjusted by Bonferroni (0.05/total SNP), but as the depth of sequencing increases, and the number of SNPs increases , many SNPs are not completely independent event. To solve this problem, some researchers to address this issue, some researchers conducted screening of population independent SNPs by LD (****reference: Association mapping for root system architecture traits under two nitrogen conditions in germplasm enhancement of maize doubled haploid lines****) to determine the threshold. In this study, we obtained 2,756,291 independent SNPs by LD screening, 0.05/2,756,291 = 1.81E-7 (-log10 [P] = 7.74). The results for the Manhattan plot showed that there were also bunches of SNPs under the threshold of 7.74. A review of previous GWAS studies in wheat found that most of the thresholds were set at 5.0 (****reference: 1. Improving grain yield, stress resilience and quality of bread wheat using large-scale genomics; 2. Genome-wide association study for resistance in bread wheat (Triticum aestivum L.) to stripe rust (Puccinia sp. tritici) races in Argentina; 3. Haplotype variations in QTL for salt tolerance in Chinese wheat accessions identified by marker-based and pedigree-based kinase and pedigree-based kinship analyses; 4. High-Resolution Genome-wide Association Study Identifies Genomic Regions and Candidate Genes for Important Agronomic Traits in Wheat Agronomic Traits in Wheat; 5. Resequencing of 145 Landmark Cultivars Reveals Asymmetric Sub-genome Selection and Strong Founder Genotype Effects on Wheat Breeding in China****), taking these considerations into account, we set the threshold for this study at 5.0. Based on the threshold of 5.0, one identified genomic region (225,328,984 bp to 225,737,586 bp) contained TaSPL17-A (TraesCS7A02G246500) and another two genes (TraesCS7A02G246400, TraesCS7A02G246300) (Fig 3a, Additional file2: Figure S5). We extracted the relative transcript levels of the three genes at six developmental stages according to inflorescence morphological features from a published transcriptome dataset of wheat spike (****reference: 1. The development of the wheat spike, 2. Genome-wide profiling of long noncoding RNAs involved in wheat spike development****).*

*On the other side, using the threshold of 7.74, we followed the screening method for GWAS candidate genes in previous publication (****reference:*** ***Resequencing of 145 Landmark Cultivars Reveals Asymmetric Sub-genome Selection and Strong Founder Genotype Effects on Wheat Breeding in China****): "****Significant P value thresholds (P <10^5^ ) were set to control the genome-wide type I error rate. Then we enlarged the candidate region to 500 kb centered on the GWAS signal peak to identity candidate genes****”. Using this method to choose candidate gene, we identified five genes in 500 kb upstream and downstream of the 7A: 225328984-225329125 (Supplement table S3), of which TraesCS7A02G246500 had the highest expression among the five genes (Supplement table S10). The results for identifying candidate genes using these two methods are the same.*

Furthermore, Bonferroni multiple test correction does not mean the division of 0.05 by the number of SNPs. It should include all tests, e.g., the number of SNPs times the number of traits in this study. The authors did not find signals on original traits of grain yield and number/size, and they derived more traits on their components. The more traits, the more associations, even by random chances. It is for sure that Bonferroni multiple test correction could be too conservative due to the linkage between SNPs and correlation among traits. However, a reasonable threshold should be provided, such as permutation.

***Authors’ response 5****: Thank you very much for the suggestion. We modified the manuscript accordingly (line 812-841 in the revised manuscript with track changes).*

*We performed permutation analysis of the 27 traits as suggested, and the main analysis steps were as follows, setting the values of each trait SEED 1-100 to obtain 100 times disrupted trait data; performing 100 GWAS analyses for each trait separately, recording the smallest P-value for each; sorting the above 100 smallest P-values from smallest to largest, choosing significance level α = 0.5, and the 0.05 quantile of the ranked P-value was used as the corrected significance level. The detailed results of the 100 runs of the traits are shown in Supplementary Table S27, with significance levels of 7.67-18.93 for 27 traits, with a threshold value of 7.67 calculated for the spikelet number trait and a threshold value of 18.93 for the middle seed weight/middle spikelet weight. We also performed Bonferroni correction of 0.05/n, where n is the number of independent markers determined by Plink (version 1.90b6.18) (window size 50000bp, step size 5, r2=0.2, n=2,756,291). This threshold of P value was determined by 0.05/2,756,291 (P=1.81e-07), –log10 [P-value] =7.74. The candidate genes in associated regions were identified and confirmed as significantly associated with the corresponding traits based on the two thresholds (–log_10_ [P value] =5.0 and 7.74), which displayed consistent results.*

If the authors have a concern about the statistical power of the study, enhancing the analytical method can make a dramatic difference. EMMAx has the same statistical power as the regular mixed linear model developed in 2005. They are both single locus models. Since then, several multiple loci models have been developed with improved statistical power, including MLMM, FarmCPU, and BLINK. The authors should focus on the improvement of statistical power instead of lowering the experiment-wise threshold without support.

***Authors’ response 6****: Thank you very much for the suggestion. As EMMAx has a limit on the number of SNPs, the software can withstand a maximum of 5 million SNPs; And the existing server memory in the laboratory is 200G, and if we want to use FarmCPU for GWAS calculations, it leads to a system crash directly after reading genotype data into R. Combining the above, we chose GCTA software for GWAS validation analysis (****Supplementary Figure 1, 2****). And the GCTA MLM analysis would be similar as that implemented in other software tools such as EMMAX, FaST-LMM and GEMMA (****reference: GCTA: a tool for genome-wide complex trait analysis; Advantages and pitfalls in the application of mixed-model association methods****).*

***Supplementary Figure 1:***  *We performed the GWAS using GAPIT software. Meanwhile, we found that we can’t loading the genotype data according the server memory in the laboratory.*

In addition to the inappropriate experiment-wise threshold, the GWAS conducted in the study also invited false positives. Figure 1C suggests that G3 and G8 are isolated from the rest groups. The GWAS section lacks details if population structure or PCs (how many) were fitted as covariates. Even if they were, it is unclear how G3 and G8 were modeled. Fitting population structures or PCs as covariates does not fully model the isolation. The dummy variables (indicators) of these two groups can remove many spurious associations with SNPs that are specific to the two groups.

Some of the traits, especially the derived traits from division, exhibit outliers which are sources of false positives. The examples include Awn wight/spile chaff weight. The outliers should be removed or pulled back to an acceptable boundary.

***Authors’ response 7****: Thank you very much for the suggestions. In the manuscript, we conducted GWAS analyses using GEMMA (version 0.98.4) software. The S matrix based on the top three PCs and the kinship (K) matrix were used for population structure correction (line 820-823 in the revised manuscript with track changes).*

*According to the suggestion, we removed the outliers and further conducnted GWAS analyses using the MLMA model of the software GCTA accordingly (****Supplementary Figure 2, middle figures****). In addition, we performed GWAS analyses of the 27 traits after removing the G3 and G8 subpopulations (****Supplementary Figure 2, middle figures****). A comparative Manhattan plot analysis of the three GWAS results for each trait revealed that the peaks of the SNPs significantly associated with the traits were constant (****Supplementary Figure 2****) between the three aproaches: the MLM model of the GEMMA software, the MLMA model of GCTA, the MLM model of the GEMMA software revoming G3 and G8 subpopulations.* ***It proved that using the first three principal components of the PCA analysis as covariates to correct the effects of population structure in this study is feasible.***

***Supplementary Figure 2: GWAS results of awn weigth and spike weight using three methods****. Top figures (a, d) show the GWAS results using MLM (PCA+Kinship) modle in GEMMA software. Middle figures (b, e) show the GWAS results using the mixed linear model based association analysis (MLMA) module in GCTA software (the outliers have been removed). Basal figures (c, f) show the GWAS results after removing the G3 and G8 subgroup result by using the same moule (MLM, PCA+kinship) and software (GEMMA).*

**Third round of review**

**Reviewer 4**

The authors have addressed all my concerns.
